# Quantifying Nutrient Profile Similarity Among Foods Using Cosine Similarity of Multidimensional Nutrient Profiles

**DOI:** 10.3390/nu18213473

**Published:** 2026-10-22

**Authors:** Renée P. M. Cardinaals, Benjamin M. Taylor, Miriam E. Clegg, Andrew J. Fletcher, Stephan Peters, Thom Huppertz

**Affiliations:** 1 Food Systems Research, Lottum, 5973 PZ Horst aan de Maas, The Netherlands; 2 School of Mathematical Sciences, University College Cork, T12 K8AF Cork, Ireland; 3 School of Food and Nutritional Sciences, University College Cork, T12 K8AF Cork, Ireland; mclegg@ucc.ie (M.E.C.); thom.huppertz@ucc.ie (T.H.); 4 Sustainable Nutrition Initiative^®^, Riddet Institute, Massey University, Palmerston North 4410, New Zealand; 5 Fonterra Research and Development Centre, Palmerston North 4472, New Zealand; 6 Dutch Dairy Association, 2509 AA Den Haag, The Netherlands; peters@nzo.nl; 7 FrieslandCampina, 3818 LE Amersfoort, The Netherlands

**Keywords:** cosine similarity, nutrient profile, nutritional metric, food substitution, healthy sustainable diets

## Abstract

Background: Food substitutions can be used for improving dietary performance in different domains, e.g., human health, environmental impact, affordability or cultural preferences. However, the criteria to justify food substitutions are often inconsistent and based on single nutrients such as maintaining volume, protein, energy or culinary equivalence. Such approaches neglect the broader and more complex nutrient profiles that define a food’s overall nutritional value. Methods: This study introduced the application of cosine similarity to enable comparisons of nutrient profiles of foods based on a multitude of nutrients as an important step in food substitutions. Each nutrient was normalized to a fraction of the Reference Daily Intake and represented an axis in a multidimensional space. Subsequently, each food in the dataset (*n* = 927) was turned into a vector in this space, and the cosine similarity between each pair of vectors was calculated. The effect of nutrient selection was assessed by using six nutrient subsets, differing from 6 to 21 essential nutrients. Results: Cosine similarity provided a quantitative measure of nutrient-profile similarity and revealed similarities across food pairs. For most food categories, we found a broad distribution of cosine similarity values, spanning from 0 to 1. The choice of nutrient subset shifted the results, highlighting the relevance of including a wide range of essential nutrients. Conclusions: We conclude that cosine similarity is a valuable tool in identifying similar nutrient profiles, offering a comprehensive and flexible framework, complementary to nutrient density approaches, for guiding dietary choices across multiple objectives without compromising nutritional quality.

## 1. Introduction

Food substitution plays a central role in contemporary nutrition science, public health guidance, and sustainability research. Individuals, institutions, and policymakers regularly make decisions that involve replacing one food with another, whether to improve human health outcomes [1,2], reduce dietary costs [1], minimize environmental impact [1,3,4,5], or accommodate cultural and personal preferences [6,7]. A food substitution approach has the benefit of being comprehensible for policymakers and consumers, while also aligning with a “small change approach” that is more feasible and maintainable compared to large dietary changes [8], and can therefore play a central role in achieving systemic changes toward more sustainable and healthy diets. At the same time, marketing claims of “healthier alternatives” require consumers to be equipped with tools that allow objective assessment of these claims. Independent of the broader objective, dietary changes through food substitutions should maintain the diet’s nutritional quality to meet human nutritional requirements, and thus consider foods’ nutritional content. To that end, many widely circulated comparisons rely on singular nutritional attributes, such as protein content [9] or caloric density [1,5,10], while neglecting the broader and more complex nutrient profiles that define a food product’s overall nutritional value. In addition, food comparisons may be kept within the boundaries of food categories, indirectly excluding the potential of food substitutions beyond food categories, and assuming food categories represent nutritional similarity [11]. This has contributed to a landscape in which food items that are nutritionally unrelated are frequently considered as though they were interchangeable, sometimes leading to distorted conclusions regarding dietary adequacy, healthfulness, or sustainability potential.

The rise in interest in plant-based diets, climate-friendly food choices, and alternative protein sources has intensified this issue. Public discourse frequently compares animal-based foods with plant-based alternatives using narrow criteria such as “grams of protein per serving” [12]. While such metrics can be useful in specific contexts, they do not capture the multidimensional nature of human nutrition, which involves a complex interaction of macronutrients, micronutrients, bioactive compounds, and, increasingly, considerations such as bioavailability. As a result, food substitutions proposed on the basis of limited nutrient metrics risk being nutritionally unbalanced and may fail to support intended dietary outcomes [13,14]. In parallel, the integration of nutrition in research on sustainability and food systems is a recent development, requiring more attention to ultimately make food comparisons that weigh in both environmental and nutritional aspects [15].

A scientifically rigorous approach for evaluating food product similarity requires acknowledging that foods exist not as single-value entities but as multidimensional nutrient vectors. Each food contains a distribution of nutrients, macronutrients, vitamins, minerals, fatty acids, and amino acids, that collectively define its role as a nutrient source in the diet. Modern food composition databases provide detailed composition data across dozens of nutrients for thousands of foods. But despite the availability of such rich data, methods for systematically comparing foods across multiple nutritional dimensions remain limited and traditional dietary analysis frameworks often stick with categorical classifications such as “protein foods,” “vegetables,” and “high-fat foods”.

Cosine similarity, a vector-based metric widely used in fields such as information retrieval, machine learning, and natural language processing, offers a promising approach for addressing this methodological gap. Although previously applied to find food substitutions for recipes based on similar ingredients [16,17], cosine similarity has not yet been applied to the analysis of food nutrient profiles. By calculating the cosine of the angle between two multidimensional vectors, the metric can capture the similarity of two nutrient profiles, independent of differences in absolute magnitude. Existing literature on nutrient profiling and diet modeling often relies on predefined scoring systems [18], distance metrics with strong magnitude dependence [17], or subjective expert classifications [19]. Nutrient density scores, which measure the contribution of a food to human nutrient requirements relative to its energy content, are among the most widely used systems to evaluate, compare and rank foods for their nutritional value. Cosine similarity, on the other hand, could evaluate the entire nutrient spectrum simultaneously and independently of portion size. That is, portion size affects the magnitude of the vector but not the relative distribution of nutrients, which makes it unique from, and complementary to, existing nutrient density approaches.

By leveraging cosine similarity, it becomes possible to evaluate whether foods commonly presented as substitutes, such as plant-based analogs for meat, grains for tubers, or nuts for dairy, are truly nutritionally comparable or whether their similarity has been overstated. Thereby, applying cosine similarity to food nutrient composition offers several advantages for both research and practical decision-making. First, it enables objective, quantitative assessment of how reasonable a proposed substitution is from a nutrient profile standpoint. Second, it minimizes the influence of anecdotal or intuitive assumptions that often drive food comparisons in public discourse and marketing. Third, it allows for systematic mapping of the “nutrient profile space” of foods, revealing clusters, gradients, and unexpected relationships across food categories, with the nutritional space being adaptable to match research objectives and data availability. Finally, it provides a tool that can complement environmental or economic analyses, allowing researchers to jointly consider nutrient profile similarity and sustainability implications in policy design or consumer guidance.

The purpose of this paper is to introduce and evaluate the use of cosine similarity as a robust quantitative framework for assessing nutrient profile similarity between foods. We apply the metric to standardized nutrient composition data to investigate patterns of similarity, identify appropriate and inappropriate substitution candidates, and highlight the extent to which common food comparisons align, or fail to align, with nutritional reality. We highlight its application with different ranges of nutrients to understand how nutrient selection impacts the results. Ultimately, cosine similarity serves as a concept that can be modified and adapted to different research aims or policy objectives, based on context-specific and the most recent data. Through this work, we aim to provide researchers, policymakers, and consumers with a scientifically grounded tool for making more informed and nutritionally coherent food substitution decisions.

## 2. Materials and Methods

### 2.1. Nutrient Composition Data and Selection of Foods

Nutrient composition data for all food items were obtained from the Dutch Food Composition database (NEVO v.2025/9.0; RIVM, Bilthoven, The Netherlands), which provides standardized nutrient profiles per 100 g of edible portion [20]. The NEVO dataset contains 2328 unique foods and nutrient profiles for over 130 nutrients, divided into 27 food categories. NEVO food categories are according to the GloboDiet system which directly aligns with the methodology applied in the Dutch National Food Consumption Survey (DNFCS) handled by the National Institute for Public Health and the Environment (RIVM) [21].For this study, we focused on essential nutrients exclusively, while nutrients to limit were not part of the analysis, as the latter are related to dietary risk factors rather than nutrient adequacy and therefore should be considered in a separate broader spectrum of dietary risk factors related to diets and foods. We extracted values for 21 essential nutrients (Table 1) for each food product in the included food categories (Table S1) for which data were available for all of these 21 essential nutrients.

From the NEVO database, a total of 927 food items were included in this study, to represent a broad range of commonly consumed items across major food categories. From the full NEVO database (*n* = 2328), alcoholic beverages (*n* = 41), miscellaneous foods (*n* = 18), herbs and spices (*n* = 51), and foods for special nutritional use (*n* = 58) were excluded as they were considered beyond the scope of this study; i.e., they are not of direct interest for food substitutions. Foods that lacked complete data for the 21 nutrients listed in Table 1 were excluded as well (*n* = 1233). The complete list of food categories and their corresponding number of food items in each category is presented in Table S1.

### 2.2. Nutrient Normalization Based on Reference Daily Intake

To contextualize nutrient content in relation to dietary requirements, we used Reference Daily Intake (RDI) values averaged for healthy adult men and women [22,23,24,25] (Table 1). RDIs were used to rescale nutrient content values to create a dimensionless nutrient axis for each food, so that differences between nutrients with varying physiological relevance or concentration ranges would not disproportionately influence similarity calculations. For each food item and each nutrient, we normalized the nutrient content per 100 g of food relative to the corresponding RDI value. Specifically, a value of 1 indicates that 100 g of the food provides 100% of the RDI for that nutrient. Values > 1 indicate that the food exceeds the RDI in 100 g, whereas values < 1 indicate lower contributions. Capping at 100% of the RDI, to avoid values > 1 indicating that a food item provides more than the RDI, was not applied. Capping is most useful when considering foods in the quantities as consumed—i.e., portion sizes—and it does not correspond to the nature of cosine similarity being independent of portion size. Furthermore, when capping is applied, a multitude of a food’s portions would also provide 100% of the RDI instead of the equivalent multitude of % RDI.

### 2.3. Cosine Similarity Calculation

Each food item was represented as a vector in a multidimensional nutritional space, where each axis corresponded to the normalized RDI-scaled content of a given nutrient. Cosine similarity was calculated using six different nutrient subsets to test the robustness of similarity estimates to the dimensionality of nutrient profiles. The analyses used defined sets of 6, 9, 11, 15, 19, or 21 nutrients (Table 1). The selection of these subsets was based on previous evaluations of nutrient density scores [26,27,28]. Subsequently, pairwise similarity between all food items was computed using the cosine similarity index, defined as:

$$\mathrm{CosineSimilarity}\left(A,B\right)=\frac{A\cdot B}{\left\|A\right\|\cdot \left\|B\right\|}$$

where *A* (*A_i_*,…,*A_p_*) and *B* (*B_i_,*…,*B_p_*) are vectors for two foods and *A*⋅*B* denotes their dot product, given by:

$$A\cdot B=\sum_{i=1}^{p}A_{i}\cdot B_{i}$$

where *A_i_* and *B_i_* denote the values of nutrient *i*, and *p* is the number of nutrients included in the analysis. ||*A*|| and ||*B*|| denote the respective magnitudes of vectors, computed as:

$$||A||=\sqrt{\sum_{i=1}^{p}A_{i}^{2}}\;\mathrm{and}\;\left\|B\right\|=\sqrt{\sum_{i=1}^{p}B_{i}^{2}}.$$

The calculated cosine similarity ranges from 0 to 1, with values approaching 1 indicating highly similar nutrient patterns and values near 0 indicating low similarity. All calculations were performed in R (version 4.5.2; Posit, Boston, MA, USA) via the RStudio Integrated Development Environment (version 2026.01.0). 

### 2.4. Analyses of Results

As food item pairs are present across food categories, cosine similarities could be assessed within (e.g., bananas compared to apples) and between food categories (e.g., bananas compared to broccoli). The analysis of the results mainly focused on the distribution of cosine similarity values, which indicates whether values were homogeneously or heterogeneously distributed within the whole dataset or within a food category. In addition, three measures were calculated to analyze the distribution of cosine similarities across the dataset: *S*10, *S*50 and *S*90 represent the 10th percentile, median and 90th percentile, respectively. Finally, the relative span (*RS*) of cosine similarities was calculated as:

$$\mathrm{RS}=\frac{\left(S90-S10\right)}{S50}$$

We applied Uniform Manifold Approximation and Projection (UMAP) in combination with cosine similarity to visualize the uniqueness of food categories’ nutrient profiles. UMAP is a dimensionality reduction tool that turns a multidimensional space into two dimensions (V1 and V2), while primarily preserving local neighborhood relationships and, to some extent, the broader structure of the data [29]. This enables the creation of a visual plot in which each data point represents a food item containing a multitude of nutrients, and the distance between data points represents their relative nutrient similarity. The x- and *y-axes* therefore do not represent actual values, as the distances between the data points are relative and not absolute. All analyses were performed in R (version 4.5.2; Posit, Boston, MA, USA) via the RStudio Integrated Development Environment (version 2026.01.0). The UMAP was created with the function umap from the R uwot package, with a minimal distance of 0.1, a number of neighbors of 15, and “cosine” as the applied metric.

## 3. Results

### 3.1. Distribution of Nutrient Vectors

**Figure 1:**
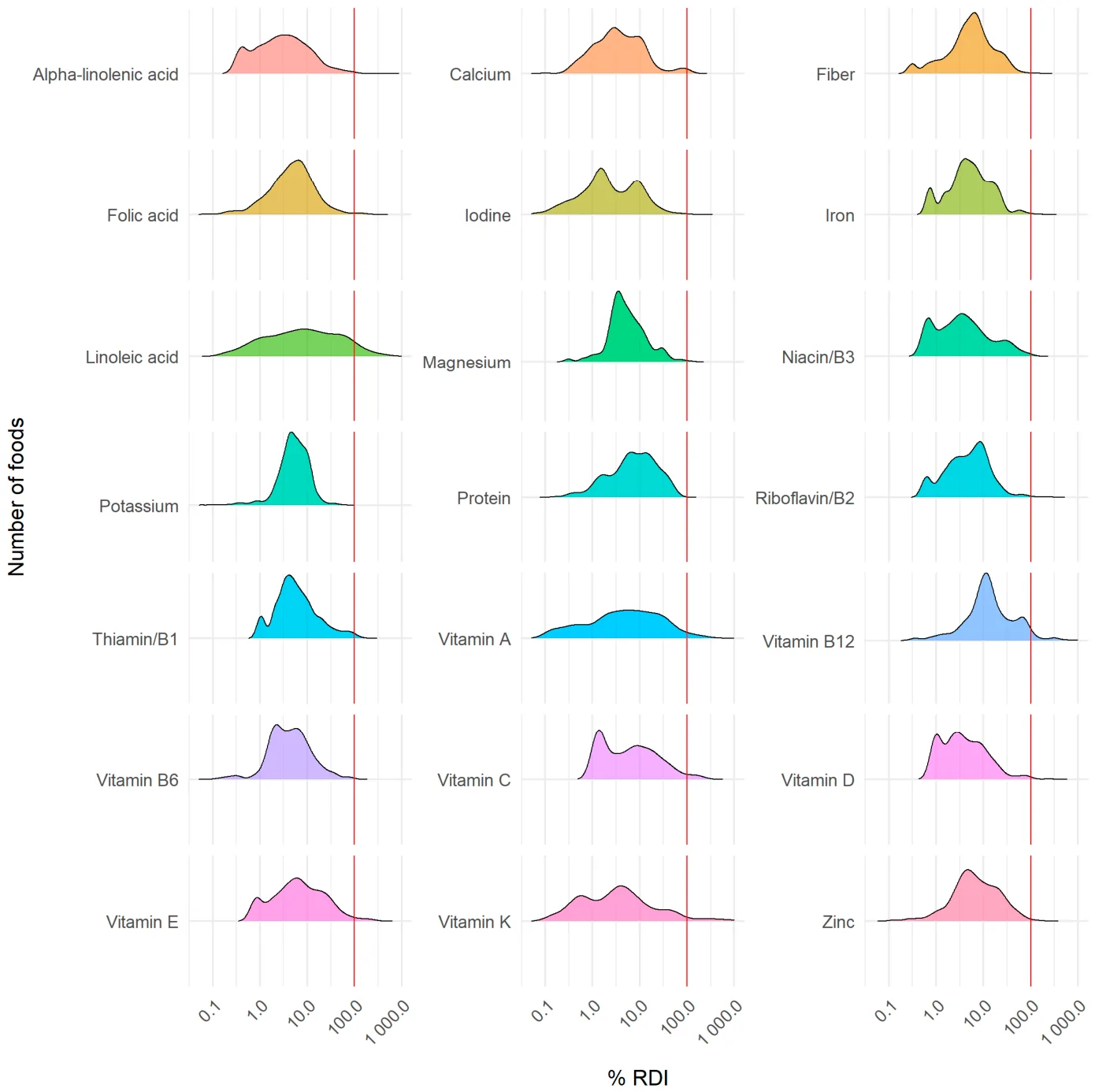
Number of foods providing a percentage of Reference Daily Intake (RDI) for the nutrients in the 21-nutrient subset (Table 1). Percentages are on a log scale; red lines indicate 100% of RDI.

Figure 1 shows, for each nutrient, how their normalized vectors were distributed among all 927 food items in the dataset. It shows that for magnesium and potassium, most foods provide between 1% and 10% of the RDI on a 100 g basis. For other nutrients, like linoleic acid or vitamin A, the nutrient content among foods has a wider range, with some foods providing < 1% of the RDI while others provide up to or above 100% of the RDI. Consequently, the choice of including or excluding such nutrients with a wide range will affect the cosine similarity values more strongly compared to nutrients with a narrower range. Furthermore, quantifying and visualizing the distribution per food category helps to understand the characteristics of a food category and can explain its (dis)similarity to other food categories (Figures S1–S21). For example, the distribution of calcium is more strongly skewed to the right (high % RDI per 100 g) for Cheese compared to Nuts and seeds, indicating dissimilarity at the food category level.

### 3.2. Cosine Similarity

Cosine similarity values were generated for all unique food combinations in the dataset (927 × 926/2) across each of the six nutrient subsets, resulting in six pairwise similarity matrices. These matrices formed the basis for all subsequent analyses of nutrient profile similarity. We first present the results for the subset of 21 nutrients, and in the next section compare those results with those obtained by applying the five other nutrient subsets.

**Figure 2:**
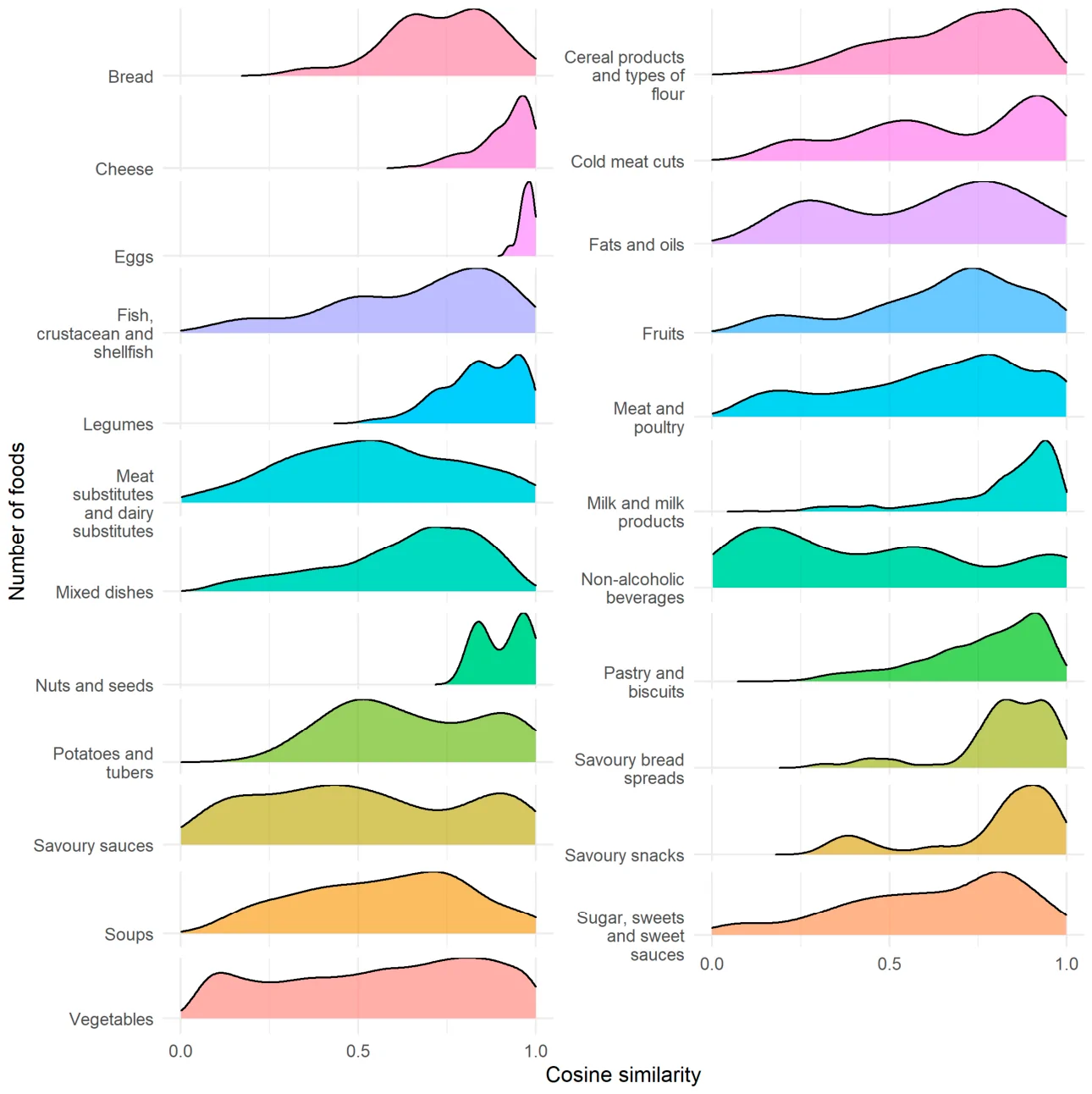
Distribution of cosine similarities, based on 21 nutrients, for combinations of foods from the same food category. Colored areas in each graph represent the number of food combinations at that cosine similarity. For the number of products in each food category, see Table S1.

Cosine similarity between food items within individual food categories, i.e., intra-category comparisons, revealed a high variation with some strong patterns (Figure 2). While some food categories show a broad range of cosine similarity values (e.g., Meat substitutes and dairy substitutes, Vegetables), others show a stronger peak towards one end of the cosine similarity range between 0 (dissimilar; e.g., Non-alcoholic beverages) and 1 (similar; e.g., Eggs, Cheese, Nuts and seeds). High variability was also reflected by S10, S50 and S90 values, with the lowest RS of 0.05 for Eggs and the highest RS of 2.25 for Non-alcoholic beverages. It should be noted that some food categories contained a low number of foods, which can also result in a low RS (Table S2).

**Figure 3:**
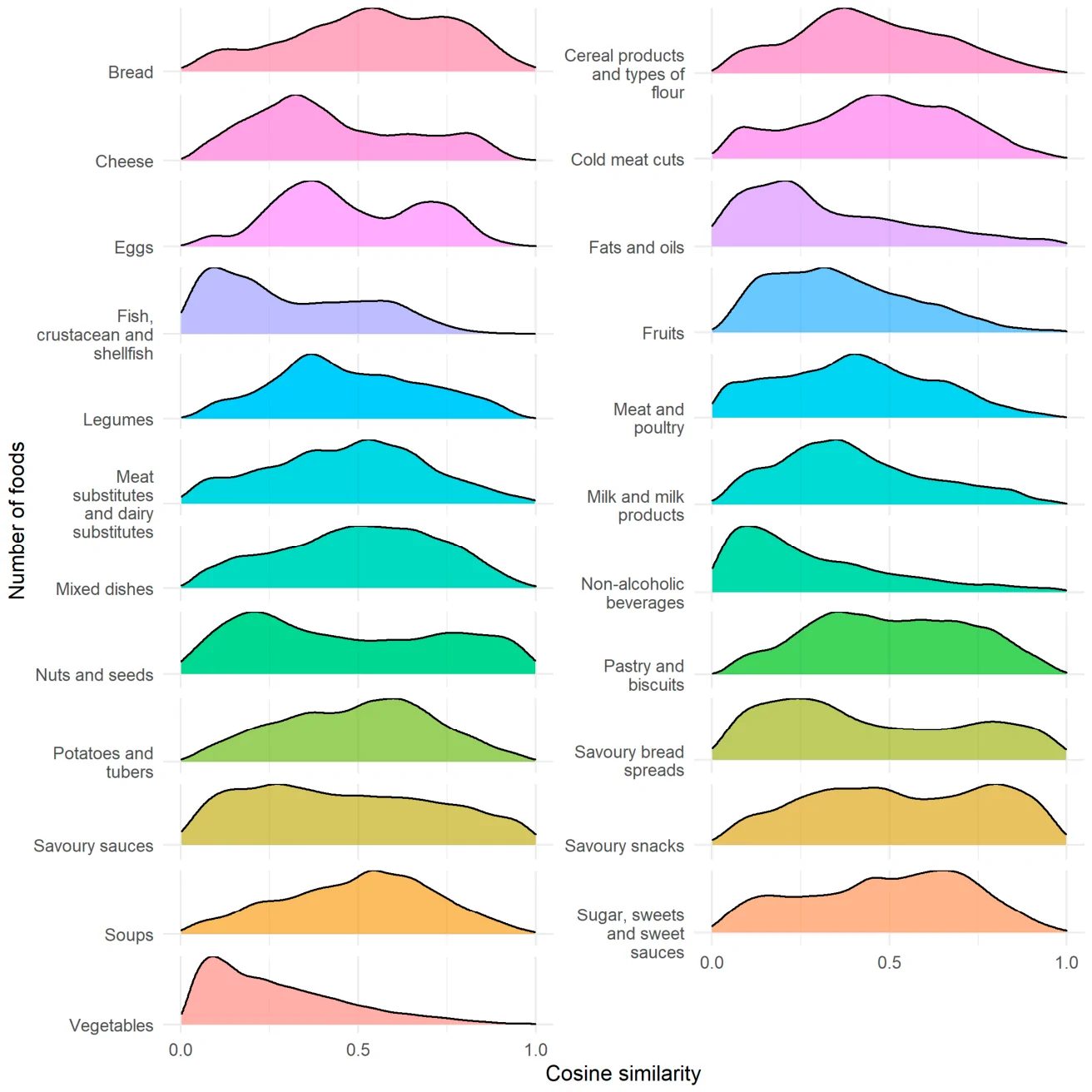
Distribution of cosine similarities, based on 21 nutrients for combinations of foods from different food categories. Colored areas in each graph represent the number of food combinations at that cosine similarity.

While Figure 2 illustrates the distribution within individual food categories, Figure 3 displays the distribution of cosine similarity values when comparing food items from different food categories (inter-category similarity). For the inter-category similarity, every single food item in the 927-product database was compared against every other product outside of its own designated food category. Consequently, the distributions in Figure 3 are notably more diverse and generally shifted toward lower similarity values compared to Figure 2. This reflects the nutritional reality that foods from different categories, such as Fish, Vegetables, or Fats and Oils, typically possess distinct nutrient profiles that make them nutritionally unique relative to the rest of the dataset. However, the inter-category analysis also reveals ‘nutritional bridges’ between seemingly unrelated groups. For instance, high cosine similarity values (>0.9) were identified between unexpected pairs, such as Brussels sprouts (Vegetables) and kiwifruits or oranges (Fruits). These findings challenge traditional culinary or material-based classifications and demonstrate how this multidimensional approach can uncover potentially similar nutrient-profile patterns, like certain grains for tubers or nuts for dairy, that might otherwise be overlooked.

### 3.3. UMAP Similarity Clusters

**Figure 4:**
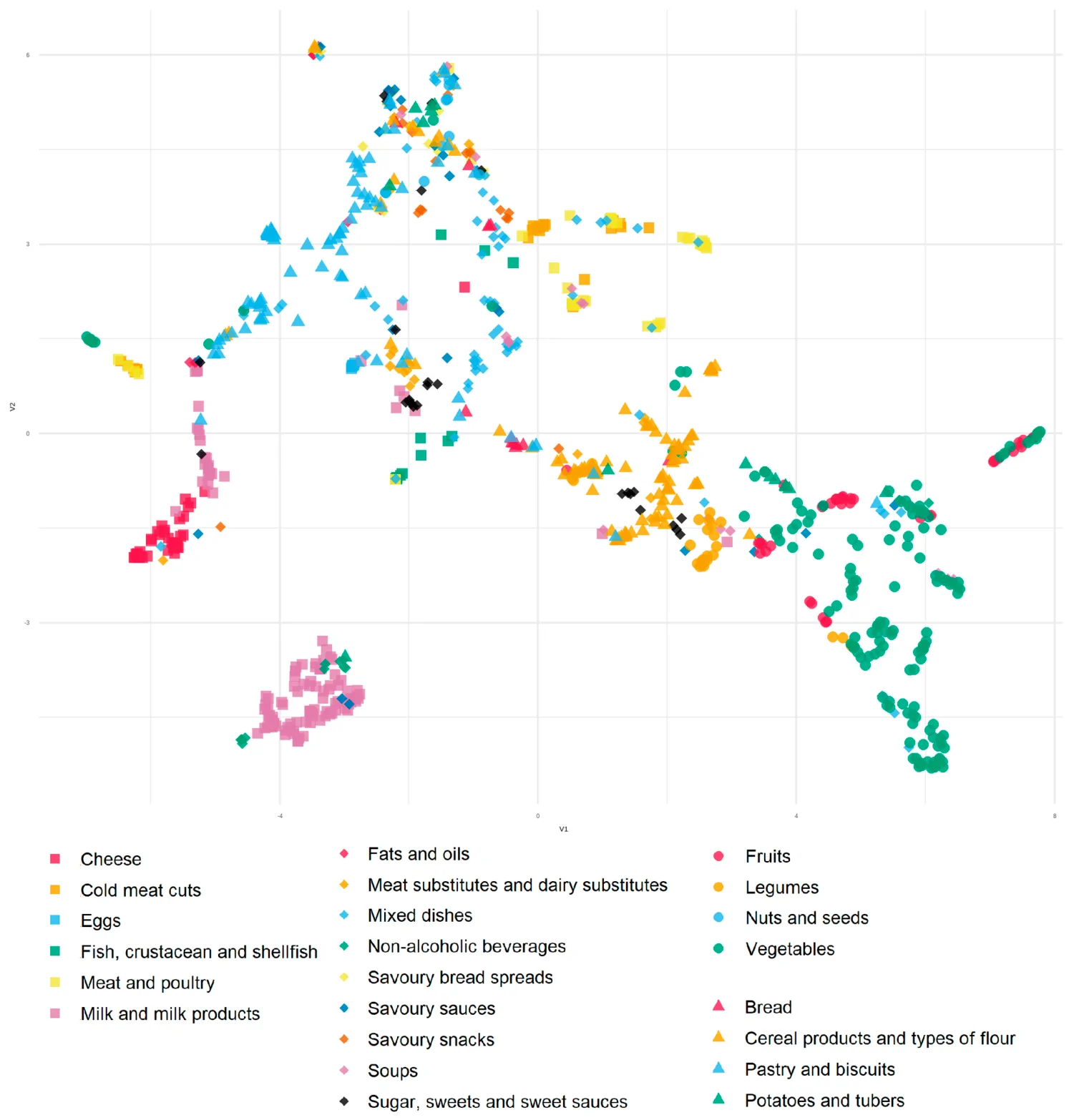
Cosine similarity of all foods in the dataset across 21 nutrients, scaled back to two dimensions using UMAP.

The UMAP reveals some similarity patterns at the level of food categories, as well as overlap and clusters of different food categories that indicate potential for substitutions with foods from different food categories (Figure 4). Food items positioned at smaller distances represent more closely related nutrient profiles. For example, products in Cheese and in Milk and milk products are strongly clustered, with these clusters also being relatively near to each other. On the other hand, some food categories are widely dispersed and occupy a broad dimensional space; i.e., it is unlikely to find substitutions between Vegetables and Pastry and biscuits.

### 3.4. Nutrient Subsets

**Figure 5:**
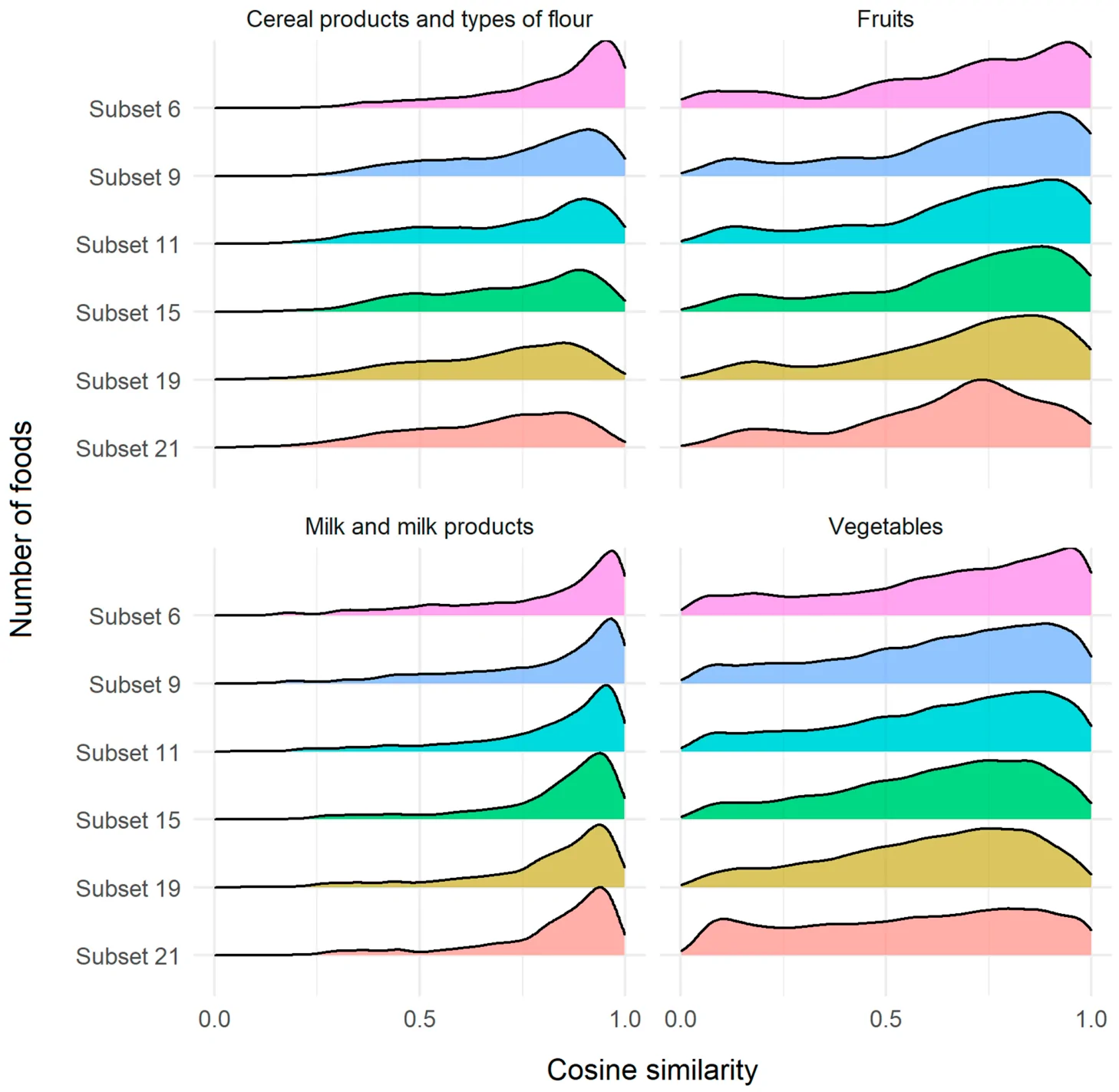
Distribution of cosine similarity values for combinations of foods from the same food category, using different nutrient subsets. Colored areas in each graph represent the number of food combinations at that cosine similarity.

The number of food combinations with high nutrient profile similarity decreases when more nutrients are included in the nutrient subset to calculate cosine similarity (Figure 5, Figures S22 and S23). Instead, with an increasing number of nutrients included in the subset, the distribution of cosine similarity becomes broader. That is, when considering more nutrients in the calculation of cosine similarity, cosine similarity of nutrient profiles tended to decrease, although for some food combinations increases were also observed. This change in average cosine similarity was observed for all food categories, but some food categories were less sensitive to changing the nutrient subset (e.g., Cheese, Milk and milk products, Pastry and biscuits). Fats and oils, on the other hand, were most sensitive to applying a higher nutrient subset as their nutrient profile was generally more monotonous with mainly high levels of vitamin E, α-linolenic acid, and linoleic acid, and including or excluding one of these had a large effect. Furthermore, which nutrient add-ons had the largest impact on similarity values differed per food category (Table 2). To illustrate, Vegetables were especially sensitive to the inclusion of vitamin K (change from 19 to 21 nutrients), while Cheese and food categories containing meat were more sensitive to the inclusion of vitamin B12 (change from 9 to 11 nutrients). This showcases how using a narrow nutrient subset may oversimplify the nutrient profile similarity, especially across different food categories.

### 3.5. Example of Cosine Similarity Application to Protein Sources

**Figure 6:**
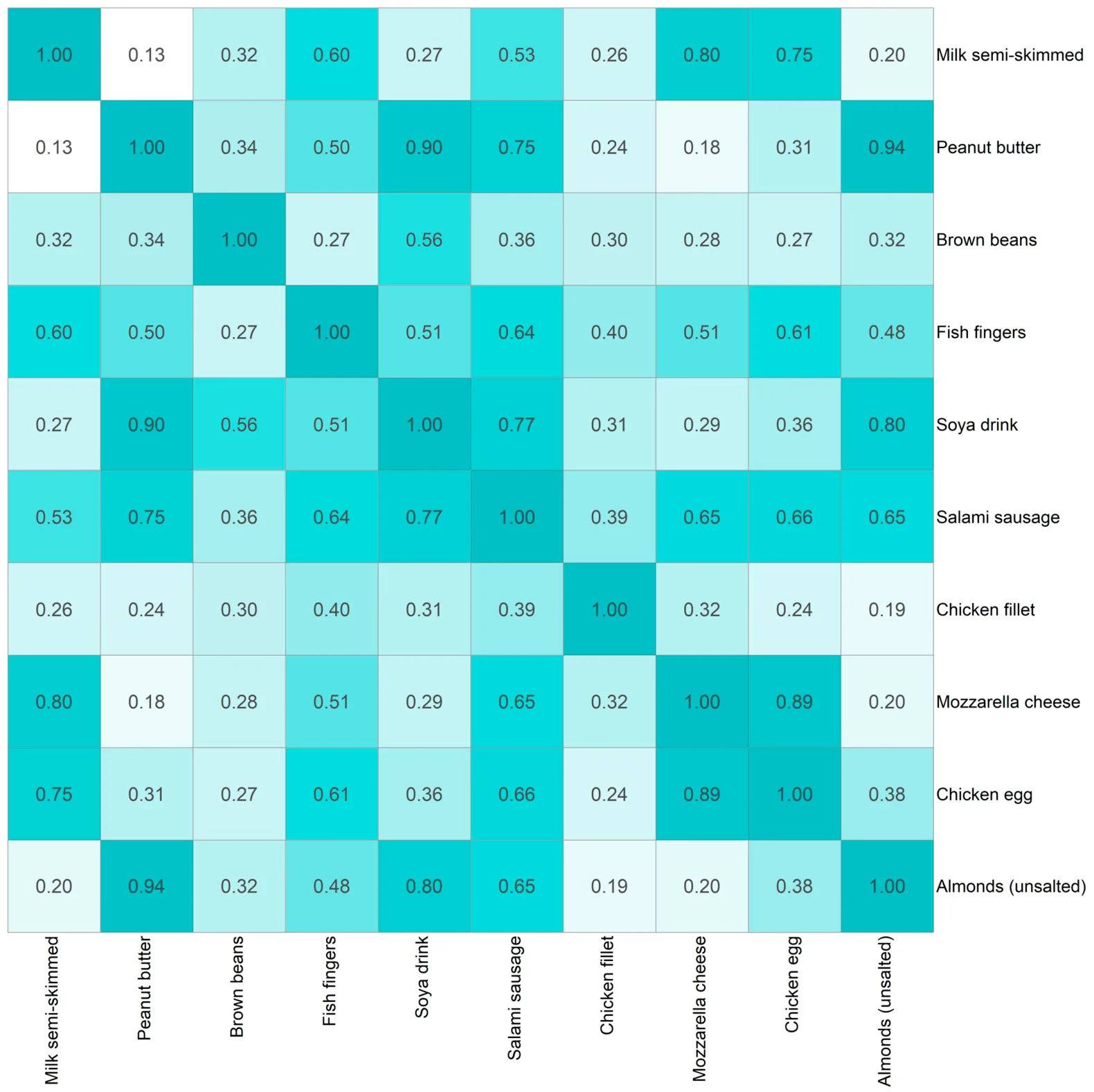
Cosine similarity matrix calculated using the 21-nutrient subset for a selection of foods categorized as “protein foods” and frequently consumed in the Netherlands. Colors indicate the strength of similarity, with darker colors being very similar and lighter colors being less similar.

To exemplify the practical application of cosine similarity, a selection of protein sources from Van Eijsden et al. [30] that represented food items most frequently consumed in the Netherlands across ten categories of protein sources was considered. When calculating the cosine similarity for all possible combinations of these food items based on their nutrient content (Table S3), a similarity matrix can be created. This matrix (Figure 6) highlights how, for these ten foods, some food combinations have a high similarity for the 21-nutrient subset, which can indicate their potential for substitutability. For example, peanut butter and soy drink show similar nutrient profiles and can therefore be candidates for substitution; this is also applicable to mozzarella cheese with either chicken eggs or semi-skimmed milk. Such a similarity matrix can also indicate which foods are more unique in their nutrient profile, in the case of a low cosine similarity with all other products (e.g., chicken fillet).

## 4. Discussion

This study demonstrates that cosine similarity provides a robust and interpretable quantitative framework for evaluating similarity of nutrient profiles across foods. Considering foods as multidimensional nutrient vectors can support moving beyond traditional single-nutrient comparisons and ambiguous categorizations and toward a more comprehensive assessment of how similar two foods truly are in terms of their nutrient profile. The results reveal clear patterns, some confirming expectations (i.e., individual foods show a broad diversity in nutritional patterns), others challenging long-held assumptions (i.e., that foods within food categories have comparable nutrient profiles), while also raising important questions about how nutrient profile similarity should be interpreted and applied in practice.

### 4.1. Interpreting Cosine Similarity in Nutritional Space

A key insight from this analysis is that nutrient profile similarity is not a binary property but exists on a continuum, and cosine similarity effectively captures this continuum. Nutrient profiling models, such as Nutrient Rich Food indices (NRF) [31], Nutri-Score [32], or the Healthy Eating Index (HEI) [33] have been developed with the aim to condense a full nutrient spectrum of a single food into one value. Thereby, nutrient profiling models are distinctly different from cosine similarity, and the two measures could be used together in complementarity. While a nutrient profiling model can, for example, show how a serving size of broccoli is as nutrient-dense as seafood or organ meat, it does not show that they exhibit a vastly different combination of nutrients. Thereby, in nutrient profiling scores, nutrients present in high concentrations may conceal nutrient gaps. Cosine similarity, on the other hand, provides a measure of the similarity in nutrient profiles individually before combining them into one value. However, while cosine similarity is a first step in looking at food substitutions, it is particularly useful when combined with information on nutrient content. Cosine similarity does not provide information about nutrient quantity, and although, e.g., Food A and Food B may contain nutrients in similar ratios, their actual content may vary between the foods. Foods that appear similar based on common public perceptions of nutrient content, such as “protein sources,” “carbohydrate staples,” or “healthy snacks”, often exhibit wide divergence in nutrient profiles, whereas some foods that are not typically thought of as comparable in terms of nutrient profiles show unexpectedly high similarity (Table S4). Even when foods are categorized into food categories, they should not be mistaken for being nutritionally similar (Table S5), and our dataset showed high variety within food categories. This underscores the need to rethink intuitive food groupings and to consider multidimensional nutrient structure rather than relying solely on categorical or cultural classifications.

### 4.2. Selection of Nutrients

**Figure A1:**
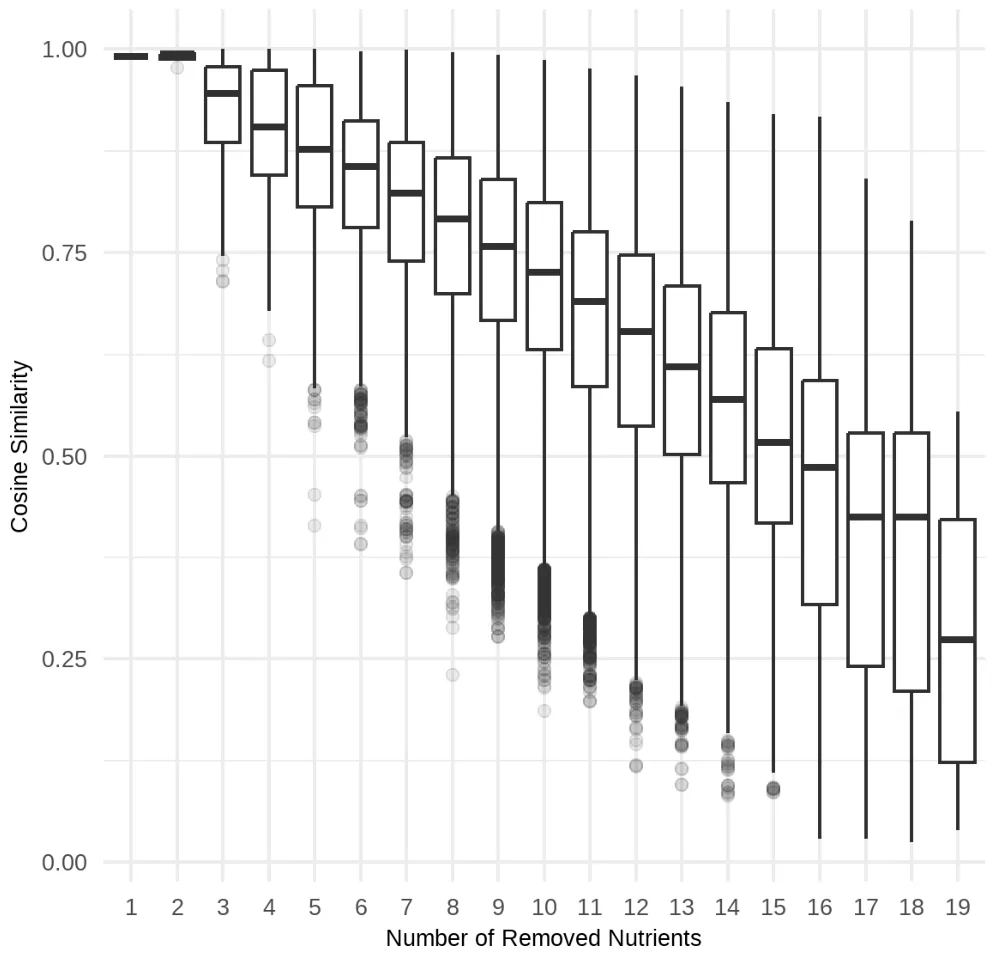
Mean and range of cosine similarity values between milk and a hypothetical product with the same nutrient content as milk while stepwise canceling out one or multiple nutrients at a time.

**Figure A2:**
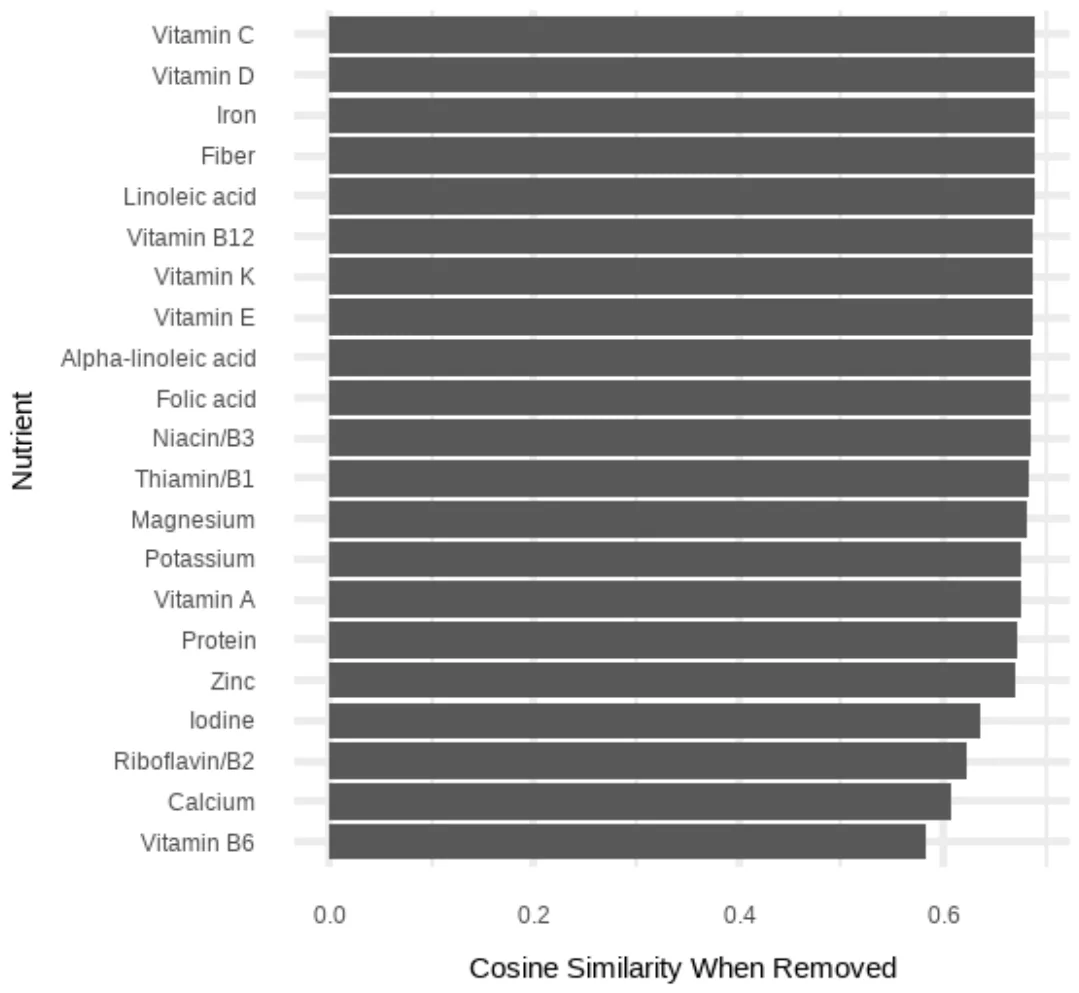
Mean cosine similarity between milk and a hypothetical product with the same nutrient content as milk while stepwise canceling out one or multiple nutrients at a time; the mean is calculated across all cosine similarity values when the considered nutrient was excluded.

A further challenge lies in the fact that cosine similarity is sensitive to dimensionality. As more nutrients are included, vectors occupy a higher-dimensional space, which changes the cosine similarity values that can be reached (Appendix A). While for some food combinations it may result in a higher similarity, for other food combinations similarity will be lower. Therefore, the selection of nutrients strongly affects the calculated cosine similarity, similar to how the selection of nutrients affects nutrient density scores [15]. This is emphasized by a comparison between two potential food substitutes, milk and a soy-based drink: a cosine similarity of 1 is calculated based on only protein, while considering a similarity of nutrient profiles based on 21 nutrients results in a cosine similarity of 0.27 (Appendix A Table A1). Similarly, when we calculated the cosine similarity for milk and a hypothetical product with the same nutrient content as milk while stepwise canceling out one or multiple nutrients at a time, the results show that cosine similarity values become more heterogeneous when compared to products with a narrow nutrient profile (Appendix A Figure A1). Particularly, this increased heterogeneity, i.e., a broader spread of cosine similarity values, is important to consider as it reflects a notable difference in contribution of individual nutrients to the calculated cosine similarity (Appendix A Figure A2).

### 4.3. Defining a Cut-Off Value for Nutrient Profile Similarity

Determining when similarity is “similar enough” for substitution or when similarity can be considered “high” or “low” is non-trivial. The metric itself provides no inherent cutoff or significance threshold, and similarity magnitudes must be interpreted relative to the empirical distribution of nutrient profiles across the dataset. The empirical distribution of the nutrient profiles reflects the properties of a larger unobserved population (i.e., a hypothetical dataset with any existing food item), of which the sample of 927 food items would be considered a random sample that represents the food items included in Dutch diets. Within the larger population of all existing food items, we would be able to calculate the exact distribution of cosine similarities between any particular food item and all others. This requires a method to address the probability that a random pair of food items would have a cosine similarity greater than or equal to a certain threshold. Because we have not observed all food items in the population, our estimate of the probability and the interval within which it lies carries uncertainty. Bootstrapping (see Appendix B) allows us to estimate this interval within the population, and our choice of threshold should take this additional variation into account. With this in mind, the cosine similarity values of 0.78 and 0.86 yielded thresholds such that, respectively, 90% and 95% of cosine similarities are below these cutoffs.

### 4.4. Implications for Nutritional Guidance and Dietary Substitution

One of the clearest applications of nutrient profile-based cosine similarity lies in evaluating the appropriateness of food substitutions, a practice that is central to diet planning, nutritional counseling, and increasingly, environmental sustainability assessments [2,3,4,5,6,7,9,10]. Many widely promoted substitutions, such as replacing animal-source foods with plant-based alternatives, or substituting one grain product for another, assume a level of nutritional equivalence that the data do not always support by using weight or portion size as the substitution unit [9]. Even substitutions based on single nutrients can lead to micronutrient deficits at the diet level [34]. Previous studies have applied cosine similarity to find food substitutions based on similarity in ingredients, but those were closely linked to food categorization [16,17], i.e., identifying foods or ingredients with similar properties such as texture, flavor, function or nutrient content, to replace foods or ingredients in a meal or recipe. At the same time, plant-based alternatives are often considered as one food category while they are in fact highly heterogeneous in nutrient profiles, both qualitatively and quantitatively [35]. Our results indicate that foods often differ across a wide range of micronutrients, even when they serve similar culinary roles or provide similar amounts of a single macronutrient such as protein or carbohydrates. Conversely, cosine similarity can highlight overlooked substitution opportunities. Based on what we observed for food categories, we hypothesize that foods that fall into different cultural or culinary categories may also share highly similar nutrient profiles and could serve as viable alternatives in nutrient-based diet modeling. Incorporating these insights could improve the design of nutritionally adequate, culturally adaptable, and economically accessible diet plans. This is particularly relevant in settings where nutrient-rich food availability or affordability is limited, such as low-income regions or humanitarian contexts, limiting the opportunities for nutritionally equal substitutions [36].

### 4.5. Applications of Cosine Similarity

#### 4.5.1. Application in Sustainable Diet Design and Environmental Assessment

Sustainability is a domain where nutrient profile-based cosine similarity offers significant value. Until recently, studies have compared foods solely based on environmental indicators, such as greenhouse gas emissions, land use, or water footprint, or based on singular nutritional indicators, such as protein content or energy [15,37]. This can lead to misleading claims for sustainable diets when foods with vastly different nutrient profiles are treated as interchangeable based on narrow metrics alone, and sustainable diets end up with incomplete nutrient profiles. The integration of environmental and nutritional aspects is a relatively new development that needs to be advanced. Although existing nutrient profiling models, such as the NRF indices, provide a metric for nutrient content or density, they do not allow for comparability among a broad variety of foods and therefore do not substantiate choices of food substitution. By complementing these metrics with a quantitative estimate of nutrient profile similarity, researchers can better align environmental assessments with the actual nutritional roles that foods play in the diet. For example, modeling climate-friendly diets based on “protein per kilogram” or “calories per hectare” may be insufficient if the foods differ substantially in micronutrient content [35]. Cosine similarity provides a means to guide environmental substitutions and limit the risk of creating micronutrient gaps [38,39]. In this context, cosine similarity can contribute to the development of nutrition-sensitive environmental assessments, bridging two fields that are often analyzed independently.

#### 4.5.2. Product Development and Reformulation

The food industry can also benefit from cosine similarity, particularly in product development and reformulation [40]. When creating alternatives designed to mimic the nutritional role of existing products, such as the design of plant-based analogs of dairy or meat, cosine similarity can serve as an objective measure of nutritional fidelity. Developers can quantify how closely a new product matches the nutrient profile of its reference product, identify specific nutrient gaps, and track improvements across successive formulations. Moreover, cosine similarity could support regulatory frameworks and labeling standards by providing transparent, reproducible criteria for when two foods can be described as nutritionally comparable.

#### 4.5.3. Public Communication and Countering Unsubstantiated Comparisons

A recurring motivation for this work is the prevalence of misleading or unsubstantiated comparisons between foods in public discourse, as well as the recurrence of misleading information and claims, or food-related “fake news”. Popular media, advocacy groups, and sometimes even scientific publications occasionally highlight contrasts based on single nutrients, such as protein or iron, or calories, that oversimplify the broader nutritional context [41]. Furthermore, there is growing concern about information and claims on food packages that can mislead consumers, often related to sugar, fat, protein or fiber content [42]. Cosine similarity offers a clear, data-driven way to counter such narratives by providing a more holistic measure of nutrient profile relatedness. It allows communicators, educators, and policymakers to demonstrate the nutritional differences in substitutions, contributing to more accurate public understanding of nutrition.

### 4.6. Methodological Considerations and Future Directions

This study was a first exploration of using cosine similarity to identify similar nutrient profiles between food items. Its practical application can benefit from further improvement by, on the one hand, exploring how other food characteristics can be included and, on the other hand, further validation of the method. While cosine similarity was shown to be powerful in the way it was formulated here, it does not capture all aspects of nutritional quality. It evaluates pattern similarity, not nutrient density, which means two foods with similar nutrient distributions but different magnitudes require additional interpretation. Future work may therefore combine cosine similarity with absolute nutrient adequacy assessments. Furthermore, the formulation and application of cosine similarity are not fixed to what is presented in this study. Instead, it is flexible and can be extended or adapted by using other nutrients or food characteristics in the mathematical computation. Promising extensions include incorporating bioavailability, amino acid profiles, and non-nutrient bioactive compounds, which are not fully represented in most standard nutrient databases or nutrient models. This implies that the cosine similarity formulation can also be adapted to the needs of a population, e.g., including nutrients for which RDIs are not met, or applying gender- or age-specific RDIs. Additionally, applying the framework to dietary patterns rather than individual foods could help identify nutritionally equivalent meal patterns or substitution pathways relevant to dietary transitions. Focusing on single substitutions could thereby help with dietary changes that are acceptable for consumers. Finally, the integration of cosine similarity with economic and environmental metrics could help establish multidimensional comparability frameworks that jointly consider nutrition, cost, and sustainability, an area of growing importance in global food system research.

## 5. Conclusions

This paper introduced the use of cosine similarity as a robust quantitative framework for assessing nutritional similarity between foods. By applying cosine similarity to a large food composition database, the main takeaways from the results were that nutrient profile similarity between individual foods exhibits a broad range, both for foods from the same food category and from different categories. Thereby, cosine similarity can reveal similar nutrient profiles that would otherwise remain undetected. Moreover, the selection of nutrients for calculating nutrient profile similarity has an impact on the final value that is not consistent across food combinations, indicating how comparisons between foods can be oversimplified when based on a narrow set of nutrients. This emphasizes how cosine similarity, with its flexibility to be adapted to the context, can benefit from including additional food aspects such as bioavailability, protein quality, or digestibility. While other tools and models focus on calculating a single value for a food’s absolute nutrient content, cosine similarity is a tool to capture the nutrient profile of two foods irrespective of the amounts that are compared. This paper illustrated how cosine similarity, as a scientifically grounded and complementary tool, can be useful for researchers, policymakers, and eventually consumers to make better-informed and nutritionally coherent food substitution decisions.

## Figures and Tables

**Table 1:** Nutrients included in the similarity calculations and their Reference Daily Intake (RDI); crosses (‘x’) indicate the inclusion of a nutrient in one of the six nutrient subsets.

	Nutrient	RDI ^1^	Nutrient Subset					
			6	9	11	15	19	21
Macronutrients	Protein	58.5 g	x	x	x	x	x	x
	Fiber	32.15 g	x	x	x	x	x	x
	Linoleic acid (LA)	5 g					x	x
	α-linolenic acid (ALA)	2.5 g					x	x
Minerals	Potassium	3500 mg		x	x	x	x	x
	Calcium	1000 mg	x	x	x	x	x	x
	Magnesium	325 mg		x	x	x	x	x
	Iron	13.5 mg	x	x	x	x	x	x
	Zinc	8 mg			x	x	x	x
	Iodine	150 µg						x
Vitamins	Vitamin A	740 µg	x	x	x	x	x	x
	Vitamin D	10 µg				x	x	x
	Vitamin K1	70 µg						x
	Vitamin E	12 µg		x	x	x	x	x
	Thiamin (B1)	0.95 mg				x	x	x
	Riboflavin (B2)	1.6 mg				x	x	x
	Niacin (B3)	15.1 mg					x	x
	Vitamin B6	1.5 mg					x	x
	Folate (B9)	300 µg				x	x	x
	Vitamin B12	2.8 µg			x	x	x	x
	Vitamin C	75 mg	x	x	x	x	x	x

^1^ RDIs were based on refs [22,23,24,25] and reflect a gender average for adults.

**Table 2:** Percentage change in average cosine similarity across all foods and within food categories when changing nutrient subsets. For nutrient subsets, see Table 1.

	Δ 6 to 9 Nutrients (%)	Δ 9 to 11 Nutrients (%)	Δ 11 to 15 Nutrients (%)	Δ 15 to 19 Nutrients (%)	Δ 19 to 21 Nutrients (%)
Average across all foods	−2.4	−1.8	−1.4	−4.5	−7.5
Bread	−9.0	−1.6	1.3	5.3	−6.0
Cereal products and types of flour	−7.9	−3.5	−1.4	−4.6	−0.8
Cheese	−0.2	−0.5	−0.8	−1.4	−0.2
Cold meat cuts	−1.1	22.6	−2.4	1.4	−0.1
Eggs	−0.5	−0.4	−0.5	0.0	−0.2
Fats and oils	37.0	−0.1	−1.3	−4.3	0.3
Fish, crustacean and shellfish	−11.7	7.9	−12.3	−10.0	−8.7
Fruits	1.4	−0.3	−0.2	−1.2	−3.9
Legumes	−1.8	0.2	−4.1	−3.7	−3.3
Meat and poultry	−2.9	−3.5	−12.8	−4.9	−0.1
Meat substitutes and dairy substitutes	−13.0	−11.9	−2.8	13.6	−1.5
Milk and milk products	0.7	1.69	0.9	−1.2	1.6
Mixed dishes	1.1	−2.62	−1.3	−2.1	−9.8
Non-alcoholic beverages	2.4	−10.94	−1.8	−1.1	1.5
Nuts and seeds	−6.3	−2.93	−3.1	8.1	−0.1
Pastry and biscuits	−0.5	−1.61	−1.9	1.7	−0.8
Potatoes and tubers	−2.9	−2.35	1.4	−12.6	−0.5
Savory bread spreads	19.1	−6.68	−0.5	12.2	−2.1
Savory sauces	−6.5	−3.71	−1.3	7.2	−4.4
Savory snacks	−1.7	−2.67	−0.2	6.7	−0.3
Soups	−4.4	−3.61	−1.8	7.0	−1.2
Sugar, sweets and sweet sauces	−4.4	−1.23	−1.2	2.1	−0.4
Vegetables	−1.9	−0.27	−1.7	−2.0	−7.5

**Figure A3:**
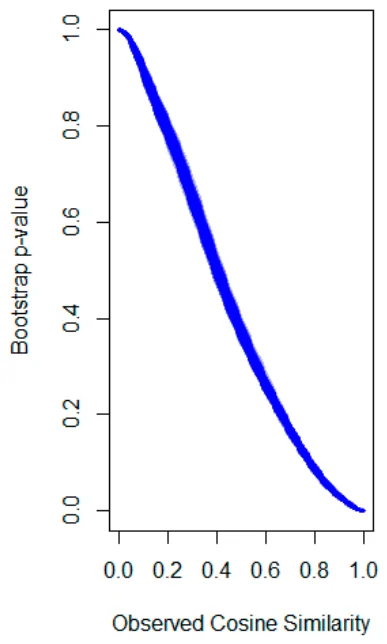
Bootstrap probabilities that observed cosine similarity exceeds the threshold, T.

## Data Availability

Nutrient content data used in this study are available in the publicly accessible database NEVO-online (https://nevo-online.rivm.nl/ (accessed on 3 August 2026)). The original contributions presented in this study are included in the article/Supplementary Materials. The code used to do the calculations will be made available upon request, and further inquiries can be directed to the corresponding author.

## Supplementary Materials

The following supporting information can be downloaded at: https://www.mdpi.com/article/10.3390/nu18213473/s1, Table S1: Food groups included in the analysis; Table S2: Average S10, S50, S90 and Relative Span per food group; Table S3: Nutrient content for food items included in Figure 6; Table S4: Food combinations for highest 50 cosine similarity values; Table S5: food combinations for lowest 50 cosine similarity values; Table S6: Cosine similarity matrix for all food combinations in this study; Figures S1–S21: Distribution of the % RDI of all 21 nutrients; Figure S22: Cosine similarity distribution for each of the six nutrient subsets for intra food category comparisons; Figure S23: Cosine similarity distribution for each of the six nutrient subsets for inter food category comparisons. Reference: [20].

## Appendix A. Impact of Nutrient Selection for Calculating Cosine Similarity

To better understand the impact of the nutrient selection for calculating cosine similarity, additional analyses were performed using cow’s milk as an example. Firstly, the cosine similarity between milk and soy drink was calculated for a single nutrient (protein) and then adding one nutrient at a time until the full subset of 21 nutrients was reached (Table A1). The results indicated how cosine similarity decreases when more nutrients are considered.

**Table A1:** Cosine similarity values between Cow’s milk and Soy drink when adding nutrients to the calculation one-by-one; values represent the nutrients already included plus the nutrient listed.

Nutrient Subset	Nutrient	%RDI per 100 g		Cosine Similarity
		Cow’s milk ^†^	Soy drink *	
6	Protein	5.8	5.8	1
	Fiber	0.0	1.9	0.95
	Calcium	12.3	1.7	0.63
	Iron	0.0	3.7	0.55
	Vitamin A	2.2	0.0	0.54
	Vitamin C	1.3	0.0	0.54
9	Potassium	4.6	4.3	0.60
	Magnesium	3.7	5.5	0.62
	Vitamin E	0.0	3.3	0.59
11	Zinc	5.1	4.4	0.64
	Vitamin B12	16.1	0.0	0.45
15	Vitamin D	0.0	0.0	0.45
	Thiamin (B1)	4.2	2.1	0.47
	Riboflavin (B2)	11.3	1.3	0.46
19	Folate (B9)	2.2	4.6	0.46
	Niacin (B3)	0.7	1.3	0.46
	Vitamin B6	2.2	3.5	0.47
	Linoleic acid (LA)	0.4	18.0	0.29
	α-linolenic acid (ALA)	0.4	4.8	0.28
21	Iodine	9.9	0.7	0.28
	Vitamin K	0.4	6.9	0.27

^†^ semi-skimmed; * without sugar, not fortified.

Secondly, the 21-nutrient subset was used as the basis, i.e., using milk’s nutrient content for the 21 essential nutrients. Subsequently, milk was compared to a hypothetical product with the same nutrient content, canceling out one nutrient at a time for each cosine similarity calculation. Thereby, the first step covered 21 cosine similarity values of milk vs. milk -1 nutrient, the second step covered 209 cosine similarity values of milk vs. milk -2 nutrients, etc. For each step, the mean cosine similarity was calculated and plotted against the number of nutrients removed (Figure A1). This showed a clear trend that the spread/heterogeneity of cosine similarity values increased when more nutrients were removed from the hypothetical product. Thus, if products tend to have a one-sided nutrient profile, cosine similarity with other products tends to be lower and more varied. Furthermore, the nutrients with the highest impact on cosine similarity could be identified, showing that for milk, at least vitamin B6, calcium and riboflavin/B2 should be included to have the most accurate representation of cosine similarity (Figure A2).

## Appendix B. Defining a Similarity Threshold

From the dataset of cosine similarity values between 927 individual foods, 100 bootstrap samples were taken. For each replicate, the empirical proportion of cosine similarity values exceeding a threshold T was calculated as the proportion of all possible pairwise cosine similarities in the bootstrap replicate. This calculation was repeated for each of the 101 potential values ranging from T = 0.00, 0.01, 0.02, …, 1.00. Across the bootstrap replicates, the empirical mean of the proportions represents the estimated probability that a cosine similarity value exceeds the threshold T in the population; we also get from this a confidence band at each threshold, as illustrated in Figure A3. The average of the curves across the bootstrap replicates was used as a lookup table.
