# Supplementary material for: Quantifying Nutrient Profile Similarity Among Foods Using Cosine Similarity of Multidimensional Nutrient Profiles
Source: Nutrients. 2026 Oct 22;18(21):3473. doi: 10.3390/nu18213473 (PMC13764778; doi:10.3390/nu18213473)
Supplement: Supplementary file 1 [file nutrients-18-03473-s001.zip › Supplementary material_1_r2.pdf]

Supplementary to

Quantifying Nutrient Profile Similarity Among Foods Using Cosine Similarity of Multidimensional Nutrient Profiles

R.P.M. Cardinaals, B. Taylor, M.E. Clegg, A.J. Fletcher, S. Peters, T. Huppertz

Table S1. Food groups included in the analysis and the number of food items in each

Food group	Number of products
Bread	12
Cereal products and types of flour	100
Cheese	38
Cold meat cuts	23
Eggs	8
Fats and oils	10
Fish, crustacean and shellfish	14
Fruits	35
Legumes	29
Meat and poultry	49
Meat and dairy substitutes	21
Milk and milk products	117
Mixed dishes	68
Non-alcoholic beverages	20
Nuts and seeds	10
Pastry and biscuits	84
Potatoes and tubers	15
Savoury bread spreads	18
Savoury sauces	28
Savoury snacks	19
Soups	18
Sugar, sweets and sweet sauces	26
Vegetables	165

Table S2. Average S10, S50, S90 and Relative Span (RS) values per food group, when cosine similarity was calculated for combinations of foods from the same food group (intra-categorical), and for combinations of foods from different food groups (inter-categorical).

Food group	S10		S50		S90		RS	
	Intra	Inter	Intra	Inter	Intra	Inter	Intra	Inter
Bread	0.54	0.15	0.76	0.49	0.90	0.78	0.48	1.29
Cereal products and types of flour	0.39	0.14	0.71	0.42	0.91	0.74	0.73	1.44
Cheese	0.79	0.14	0.93	0.37	0.99	0.78	0.22	1.75
Cold meat cuts	0.26	0.12	0.70	0.46	0.96	0.74	1.00	1.36
Eggs	0.95	0.20	0.97	0.44	1.00	0.76	0.05	1.29
Fats and oils	0.25	0.05	0.71	0.24	0.89	0.70	0.90	2.71
Fish, crustacean and shellfish	0.30	0.06	0.74	0.27	0.90	0.66	0.81	2.24
Fruits	0.26	0.12	0.70	0.34	0.93	0.66	0.95	1.58
Legumes	0.70	0.21	0.85	0.43	0.97	0.75	0.31	1.27
Meat and poultry	0.21	0.09	0.68	0.40	0.96	0.69	1.10	1.53
Meat and dairy substitutes	0.24	0.10	0.54	0.41	0.88	0.73	1.19	1.52
Milk and milk products	0.57	0.12	0.88	0.37	0.97	0.71	0.45	1.61
Mixed dishes	0.31	0.19	0.68	0.50	0.88	0.79	0.84	1.19
Non-alcoholic beverages	0.09	0.06	0.39	0.26	0.97	0.67	2.25	2.36
Nuts and seeds	0.82	0.09	0.92	0.37	0.99	0.85	0.18	2.08
Pastry and biscuits	0.51	0.16	0.80	0.45	0.94	0.78	0.54	1.37
Potatoes and tubers	0.38	0.16	0.61	0.47	0.95	0.76	0.93	1.27
Savoury bread spreads	0.62	0.10	0.85	0.33	0.97	0.81	0.41	2.18
Savoury sauces	0.13	0.10	0.49	0.37	0.93	0.80	1.63	1.92
Savoury snacks	0.41	0.12	0.86	0.43	0.96	0.85	0.65	1.66
Soups	0.24	0.17	0.58	0.50	0.85	0.76	1.05	1.19
Sugar, sweets and sweet sauces	0.27	0.12	0.66	0.47	0.90	0.77	0.96	1.37
Vegetables	0.13	0.06	0.59	0.24	0.92	0.58	1.33	2.13

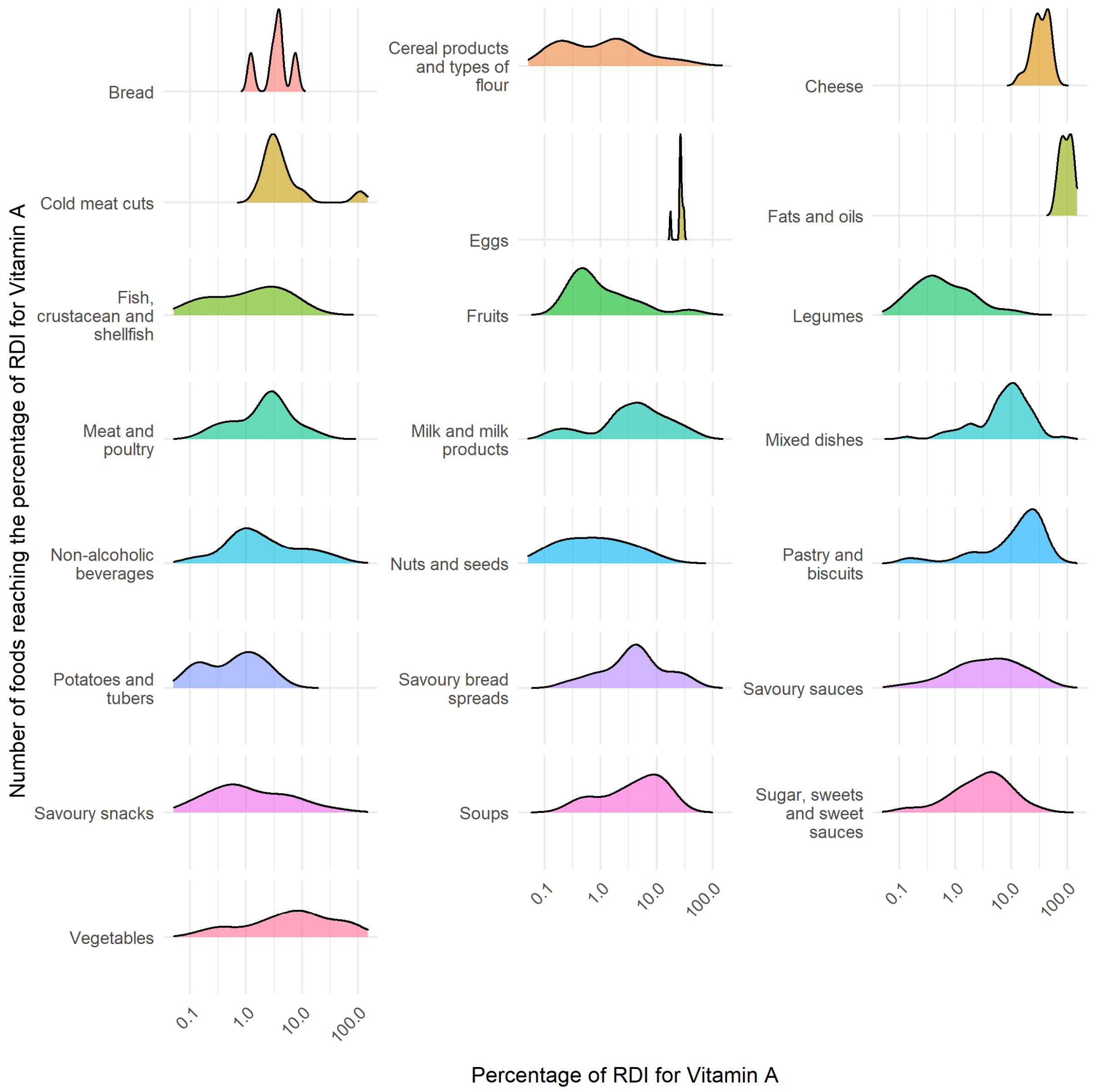

Figure S1. Distribution of the % of Reference Daily Intake (RDI) of vitamin A provided by 100 g of all food products belonging to one food group. The food group "Meat substitutes and dairy substitutes" is not shown because vitamin A content was (close to) 0 for all foods included in this group.

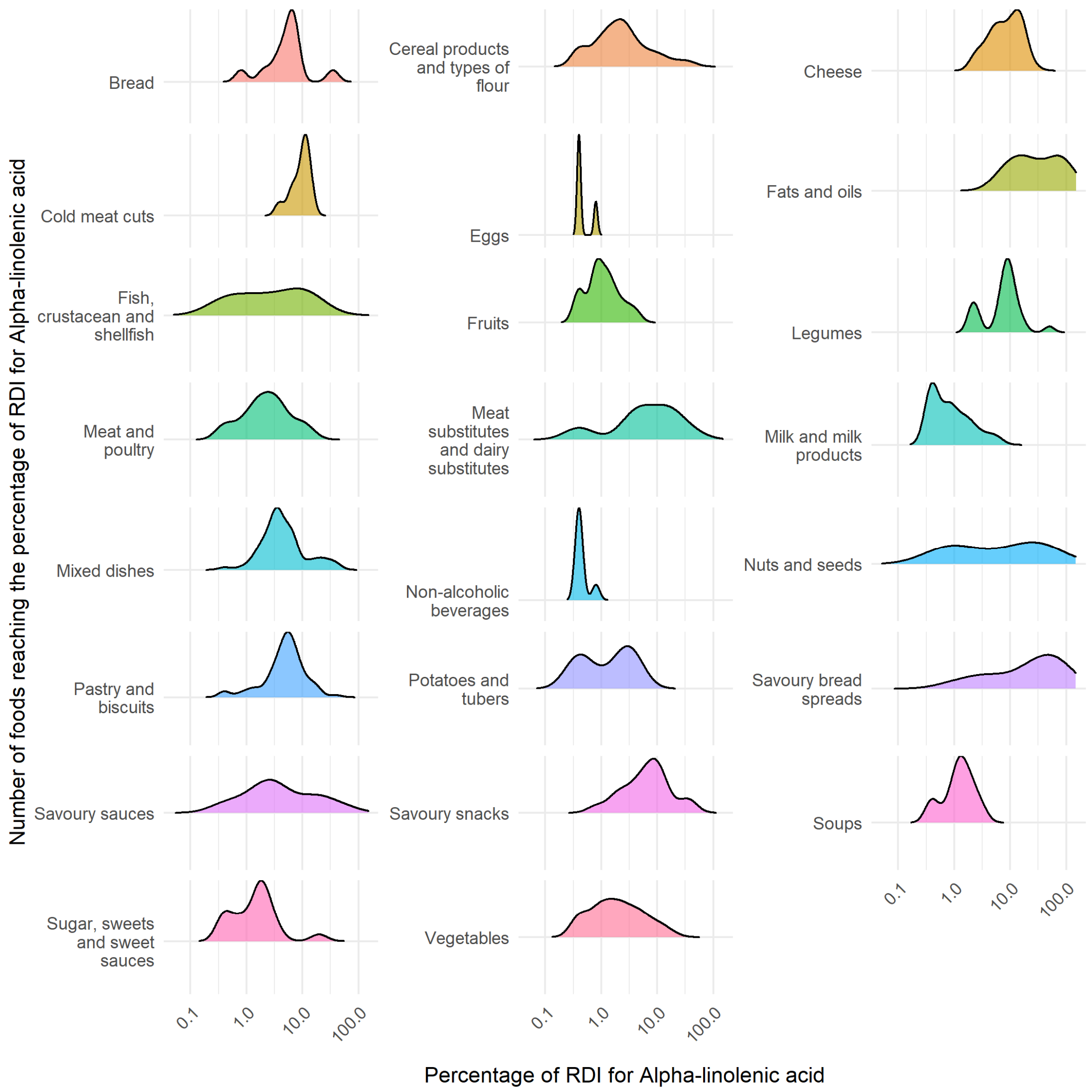

Figure S2. Foods contributing to Reference Daily Intake (RDI) of alpha-linolenic acid per 100 gram for each food group; coloured area indicates the amount of foods providing the subsequent percentage of RDI.

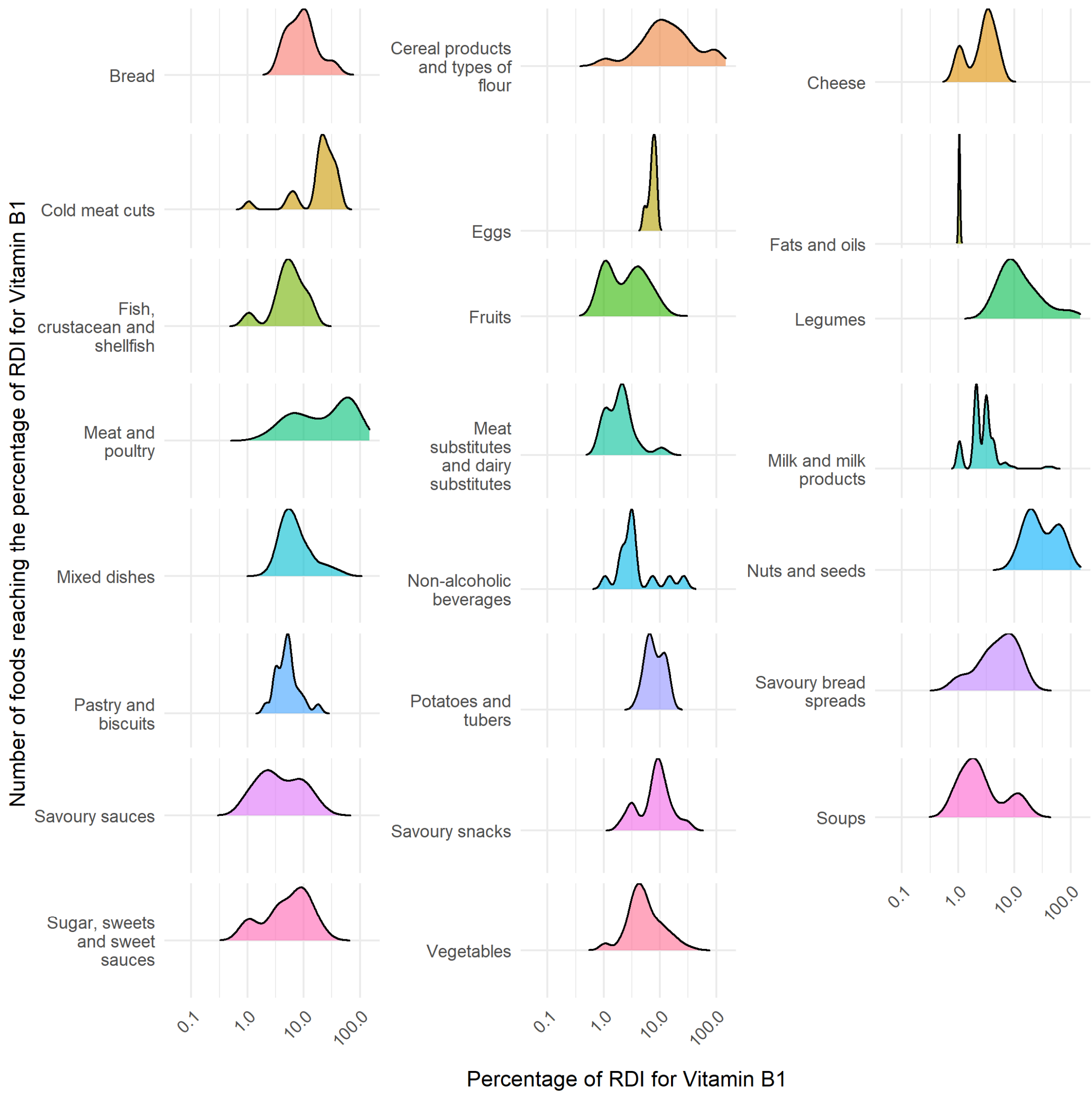

Figure S3. Foods contributing to Reference Daily Intake (RDI) of Vitamin B1 (Thiamin) per 100 gram for each food group; coloured area indicates the amount of foods providing the subsequent percentage of RDI.

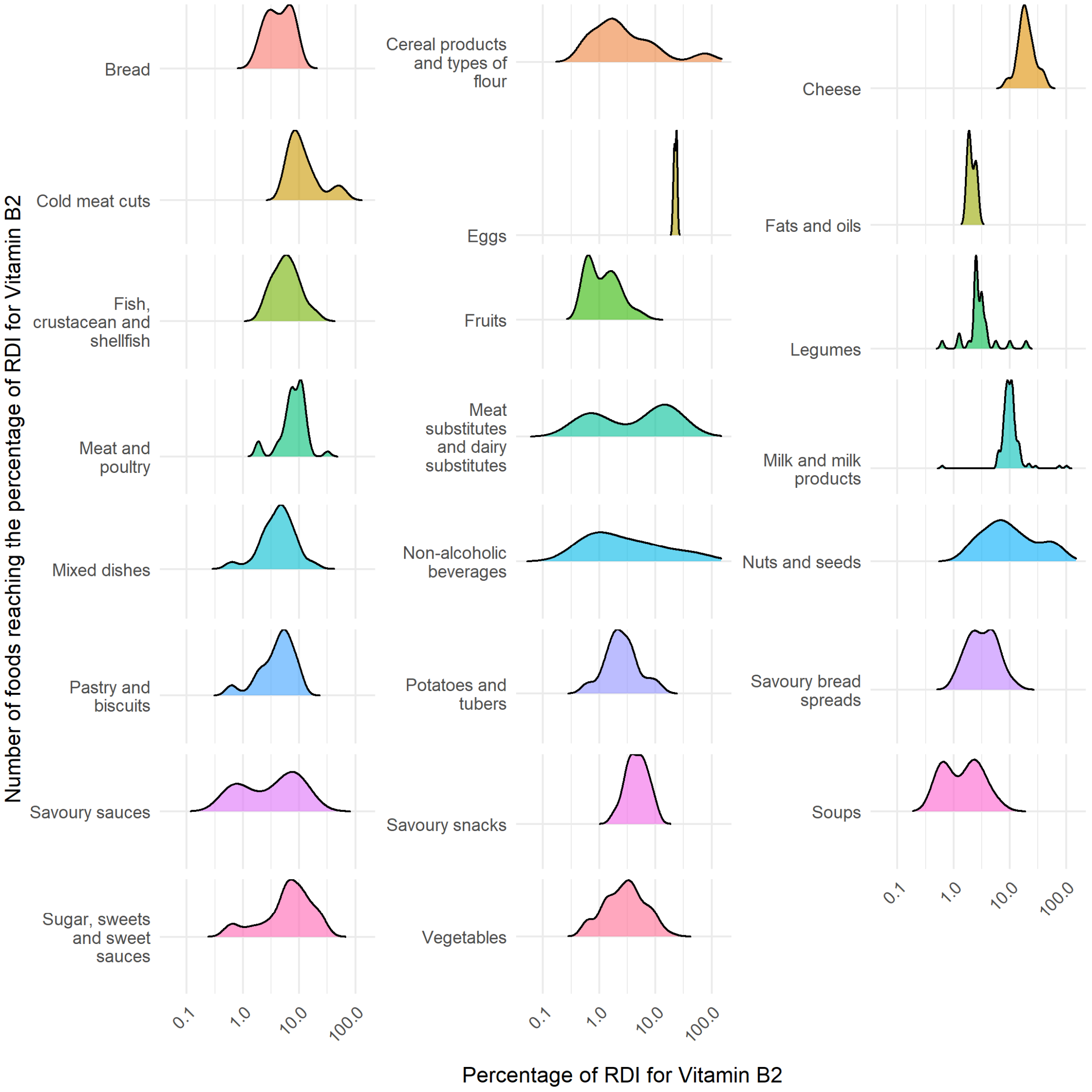

Figure S4. Foods contributing to Reference Daily Intake (RDI) of Vitamin B2 (Riboflavin) per 100 gram for each food group; coloured area indicates the amount of foods providing the subsequent percentage of RDI.

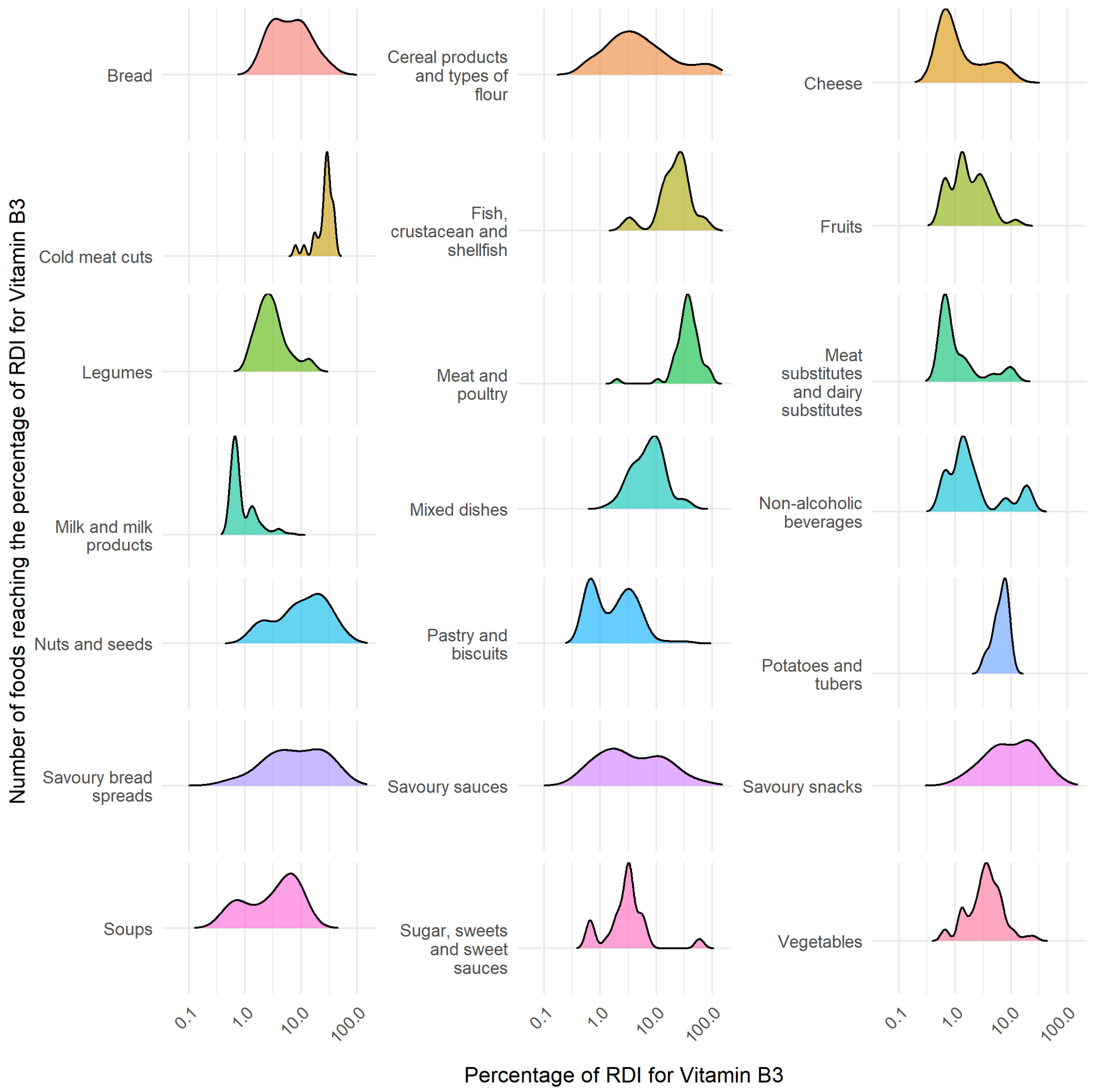

Figure S5. Foods contributing to Reference Daily Intake (RDI) of Vitamin B3 (Niacin) per 100 gram for each food group; coloured area indicates the amount of foods providing the subsequent percentage of RDI.

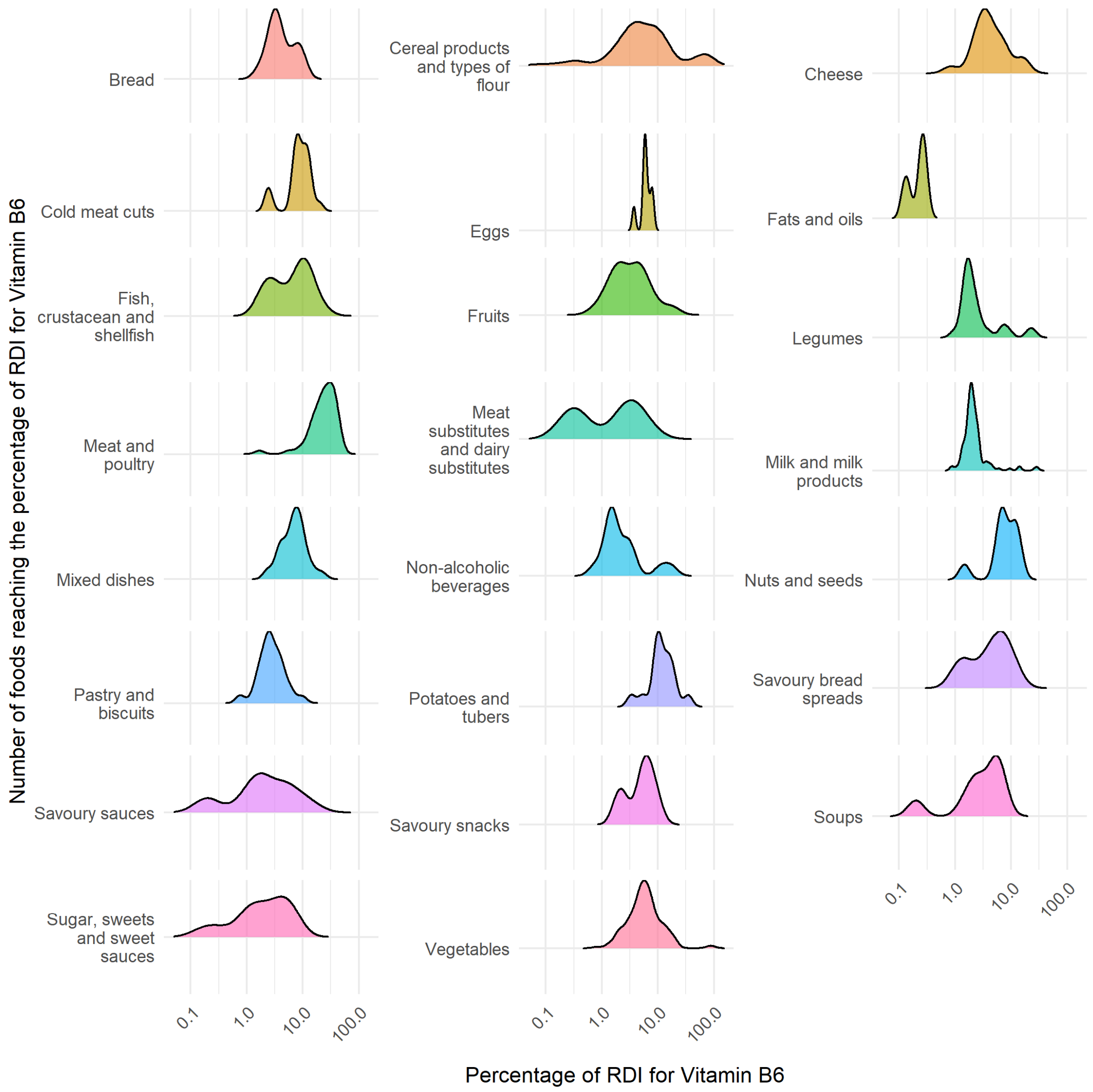

Figure S6. Foods contributing to Reference Daily Intake (RDI) of Vitamin B6 per 100 gram for each food group; coloured area indicates the amount of foods providing the subsequent percentage of RDI.

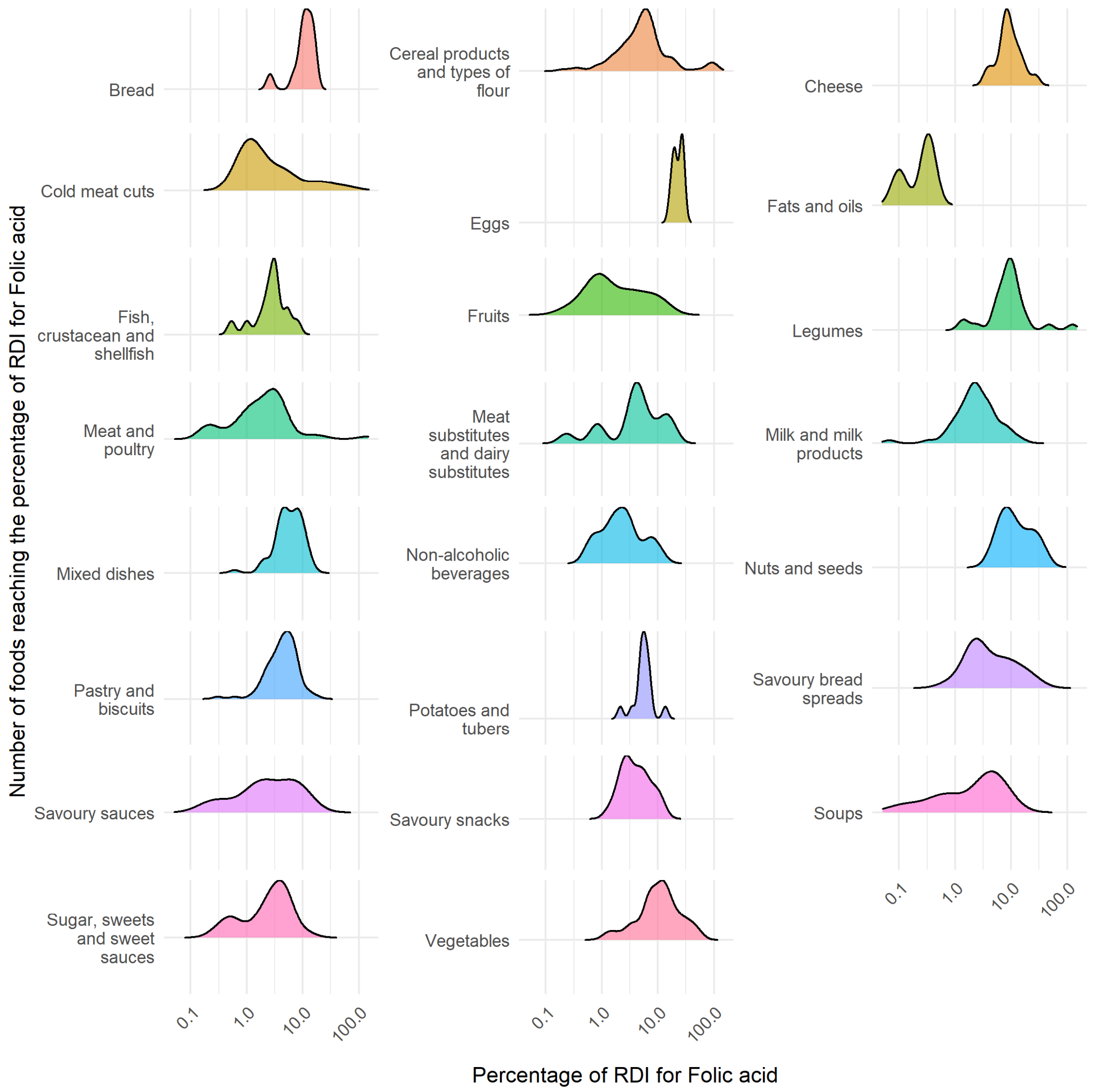

Figure S7. Foods contributing to Reference Daily Intake (RDI) of Folic acid (B9) per 100 gram for each food group; coloured area indicates the amount of foods providing the subsequent percentage of RDI.

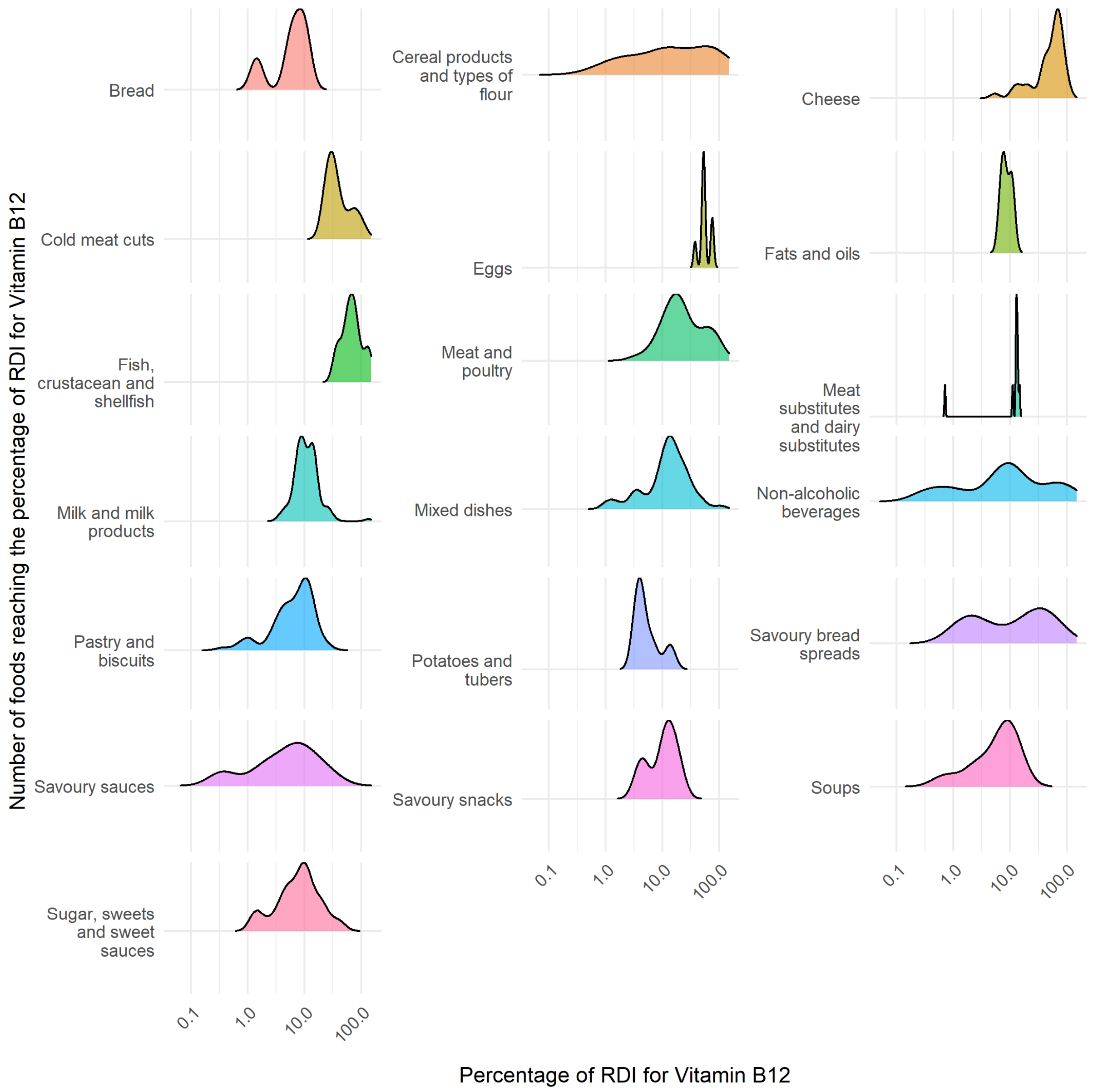

Figure S8. Foods contributing to Reference Daily Intake (RDI) of Vitamin B12 per 100 gram for each food group; coloured area indicates the amount of foods providing the subsequent percentage of RDI.

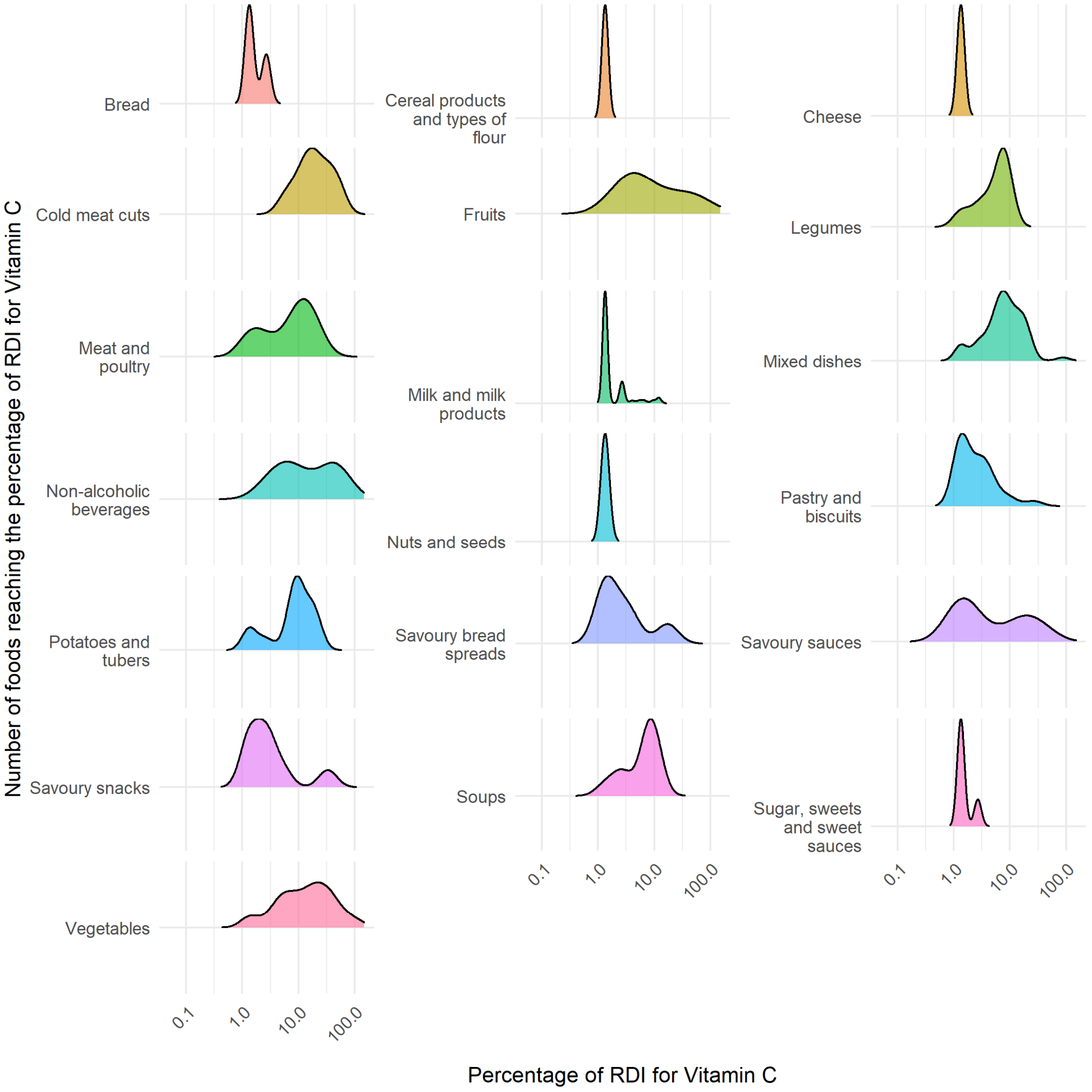

Figure S9. Foods contributing to Reference Daily Intake (RDI) of Vitamin C per 100 gram for each food group; coloured area indicates the amount of foods providing the subsequent percentage of RDI.

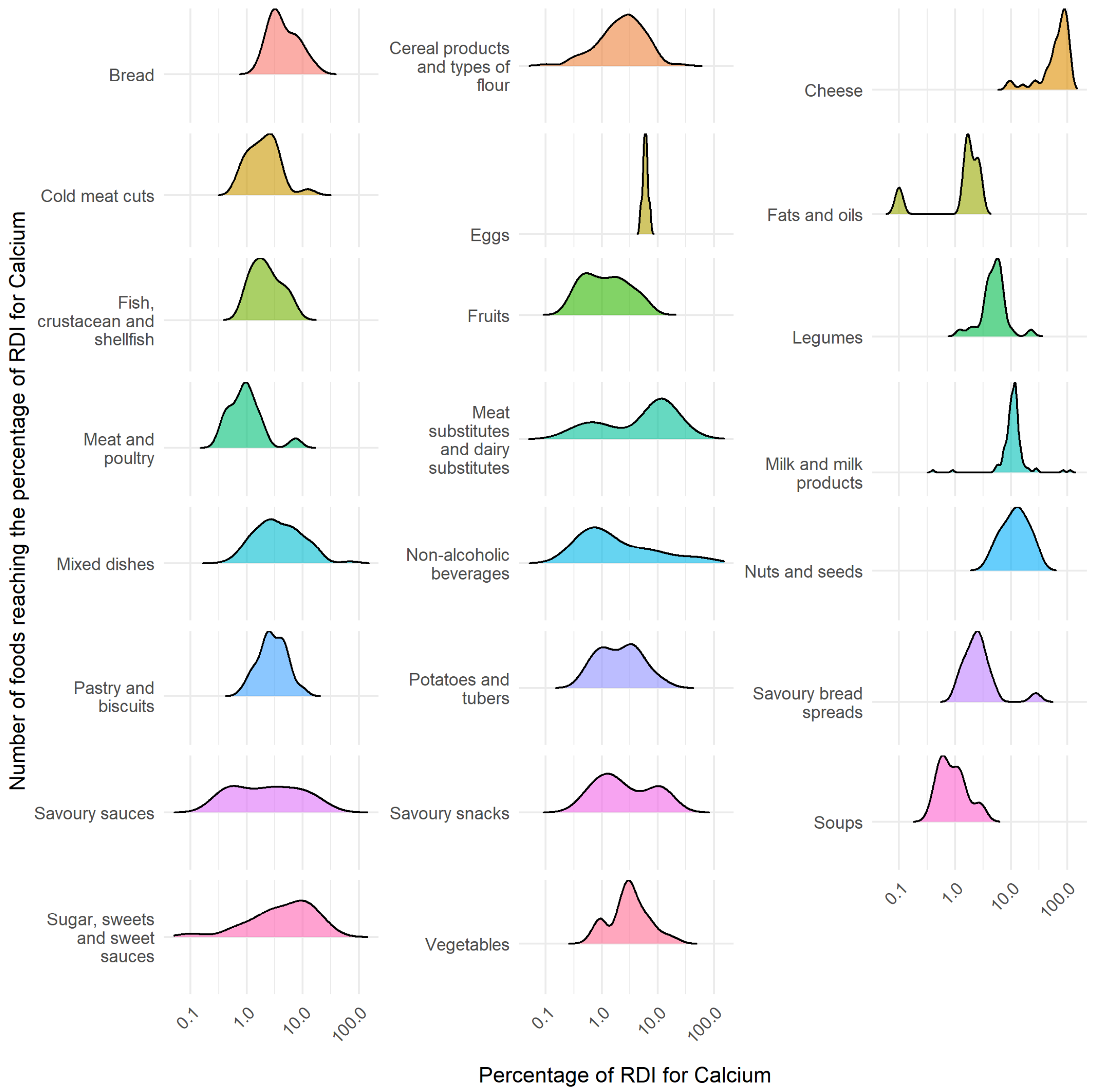

Figure S10. Foods contributing to Reference Daily Intake (RDI) of Calcium per 100 gram for each food group; coloured area indicates the amount of foods providing the subsequent percentage of RDI.

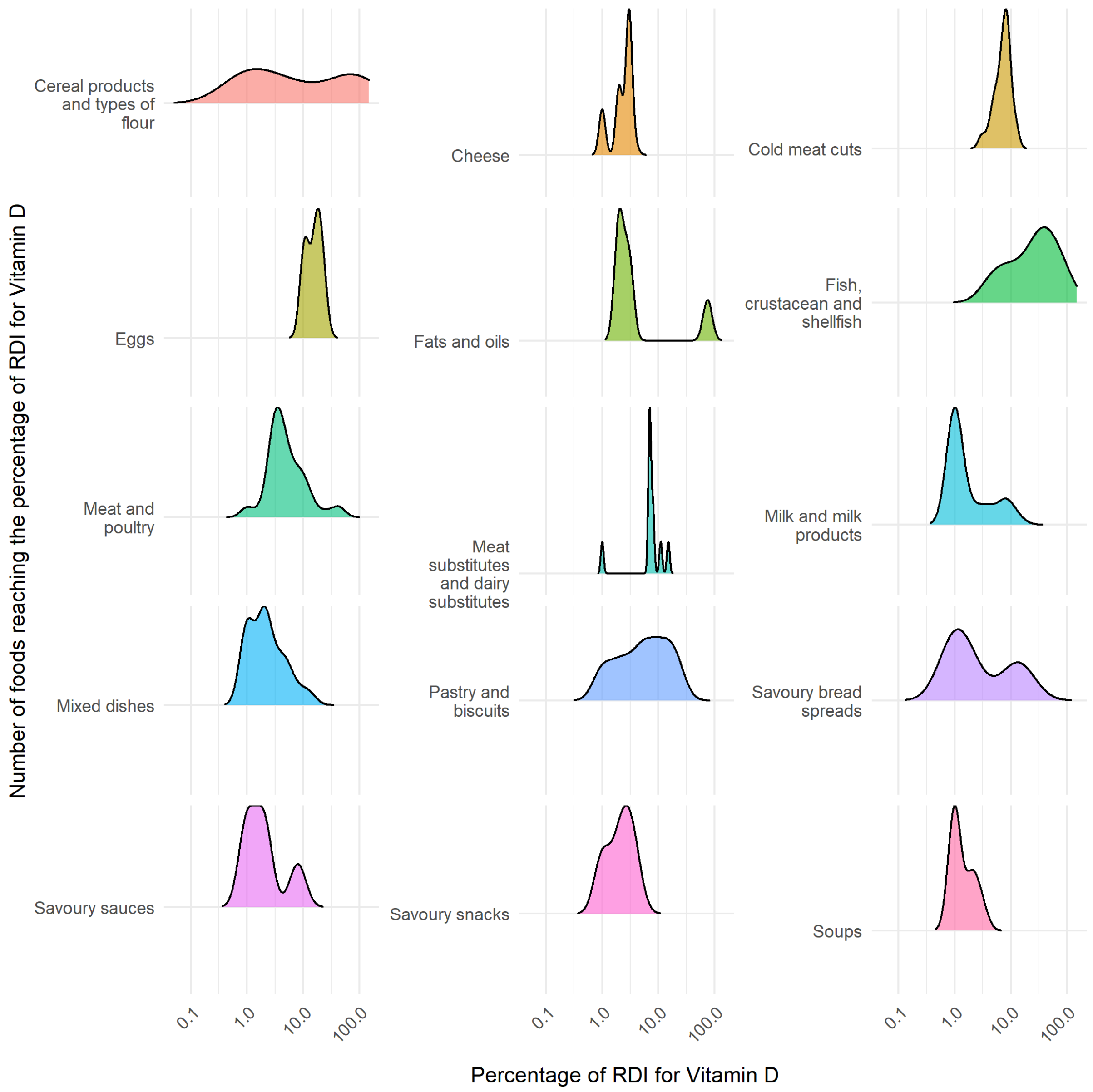

Figure S11. Foods contributing to Reference Daily Intake (RDI) of Vitamin D per 100 gram for each food group; coloured area indicates the amount of foods providing the subsequent percentage of RDI.

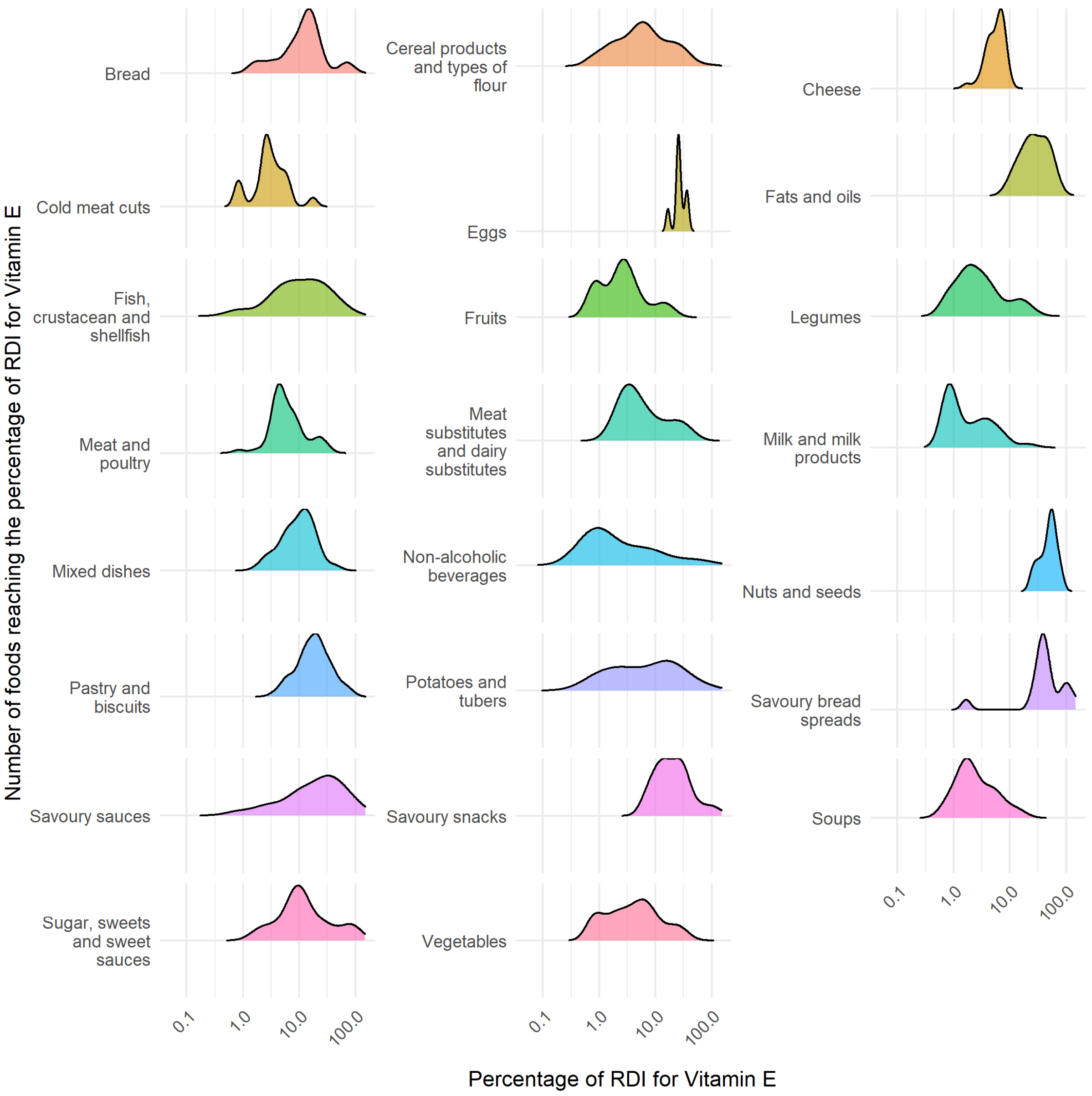

Figure S12. Foods contributing to Reference Daily Intake (RDI) of Vitamin E per 100 gram for each food group; coloured area indicates the amount of foods providing the subsequent percentage of RDI.

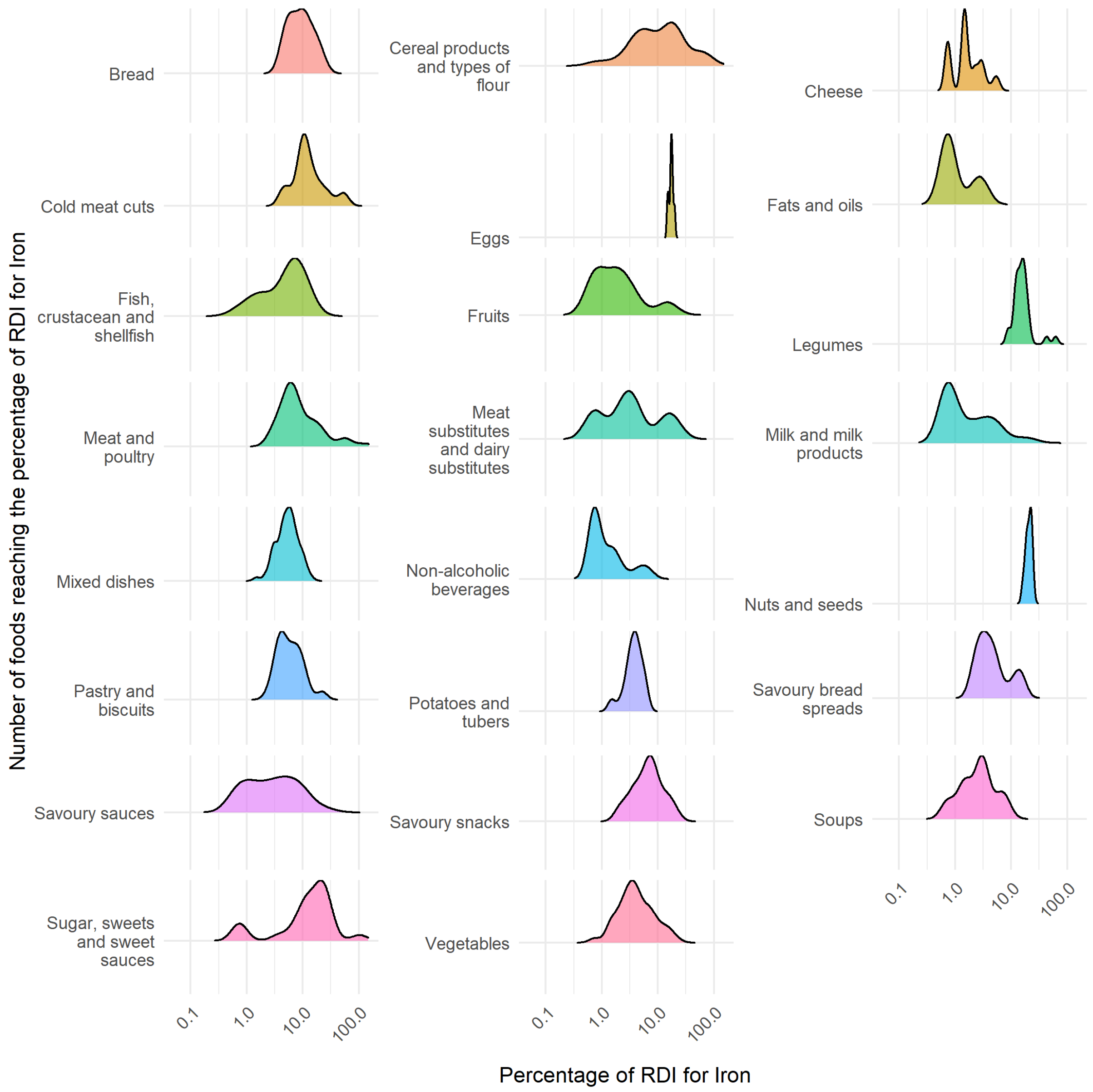

Figure S13. Foods contributing to Reference Daily Intake (RDI) of Iron per 100 gram for each food group; coloured area indicates the amount of foods providing the subsequent percentage of RDI.

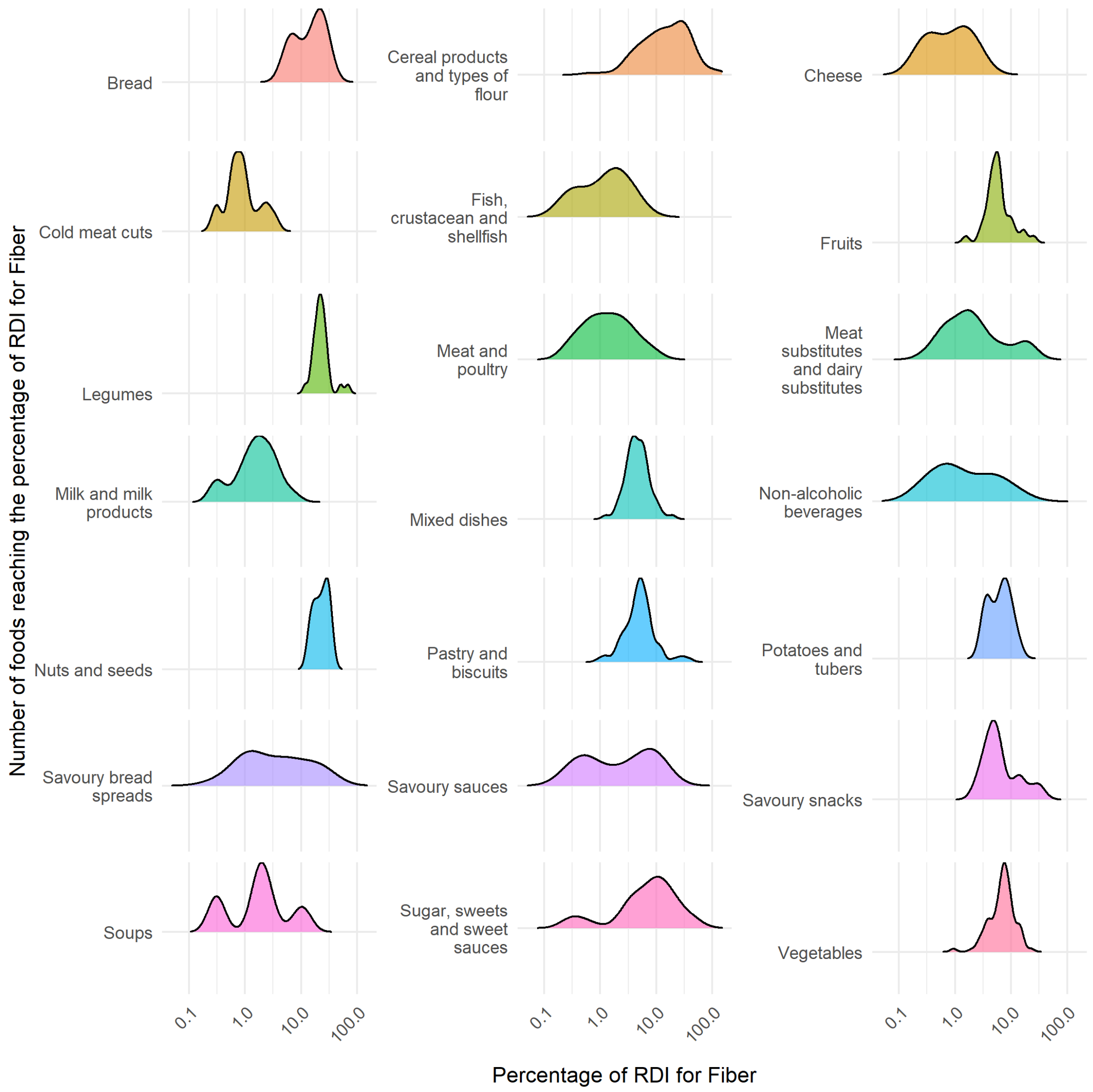

Figure S14. Foods contributing to Reference Daily Intake (RDI) of Fiber per 100 gram for each food group; coloured area indicates the amount of foods providing the subsequent percentage of RDI.

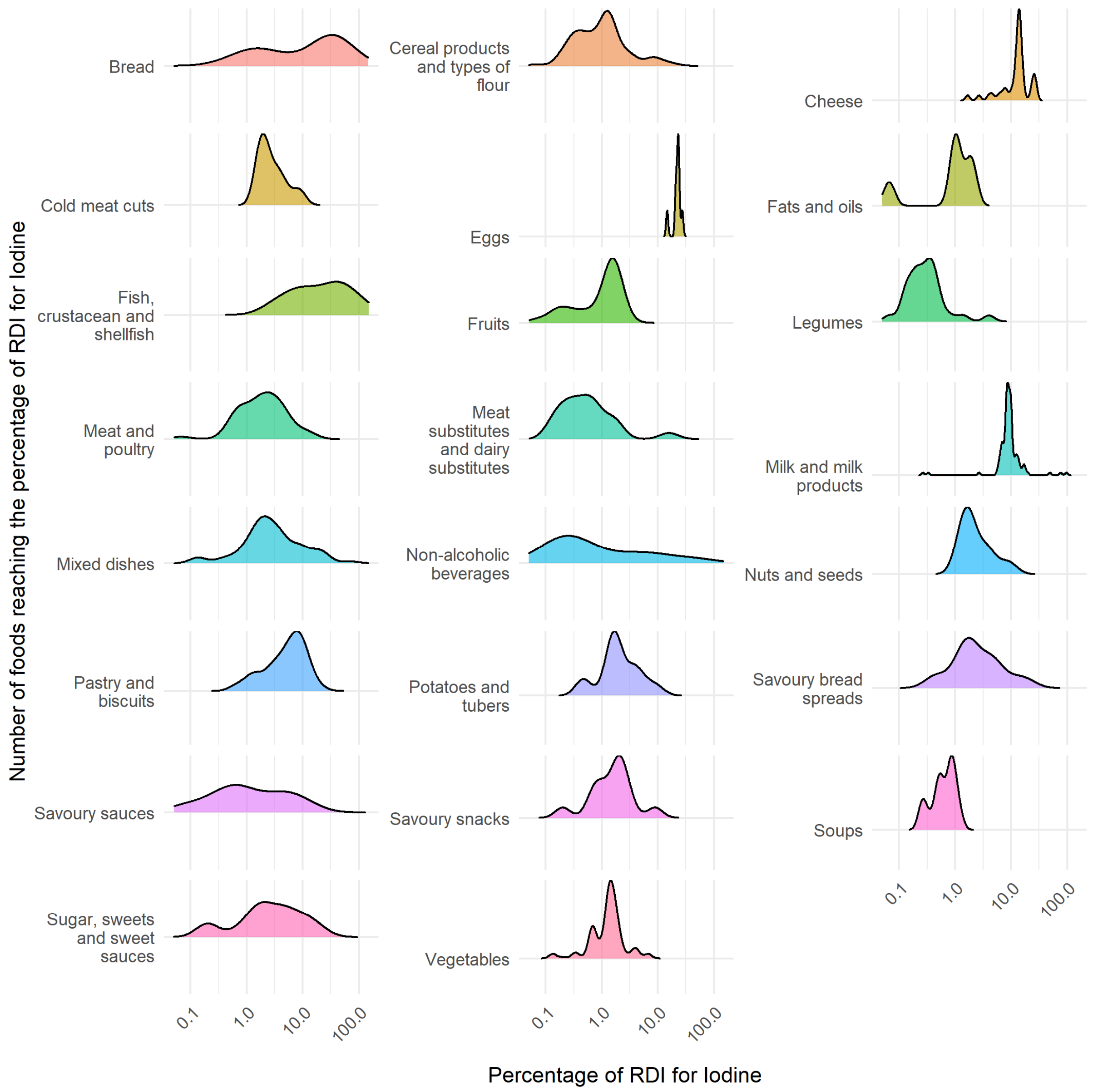

Figure S15. Foods contributing to Reference Daily Intake (RDI) of Iodine per 100 gram for each food group; coloured area indicates the amount of foods providing the subsequent percentage of RDI.

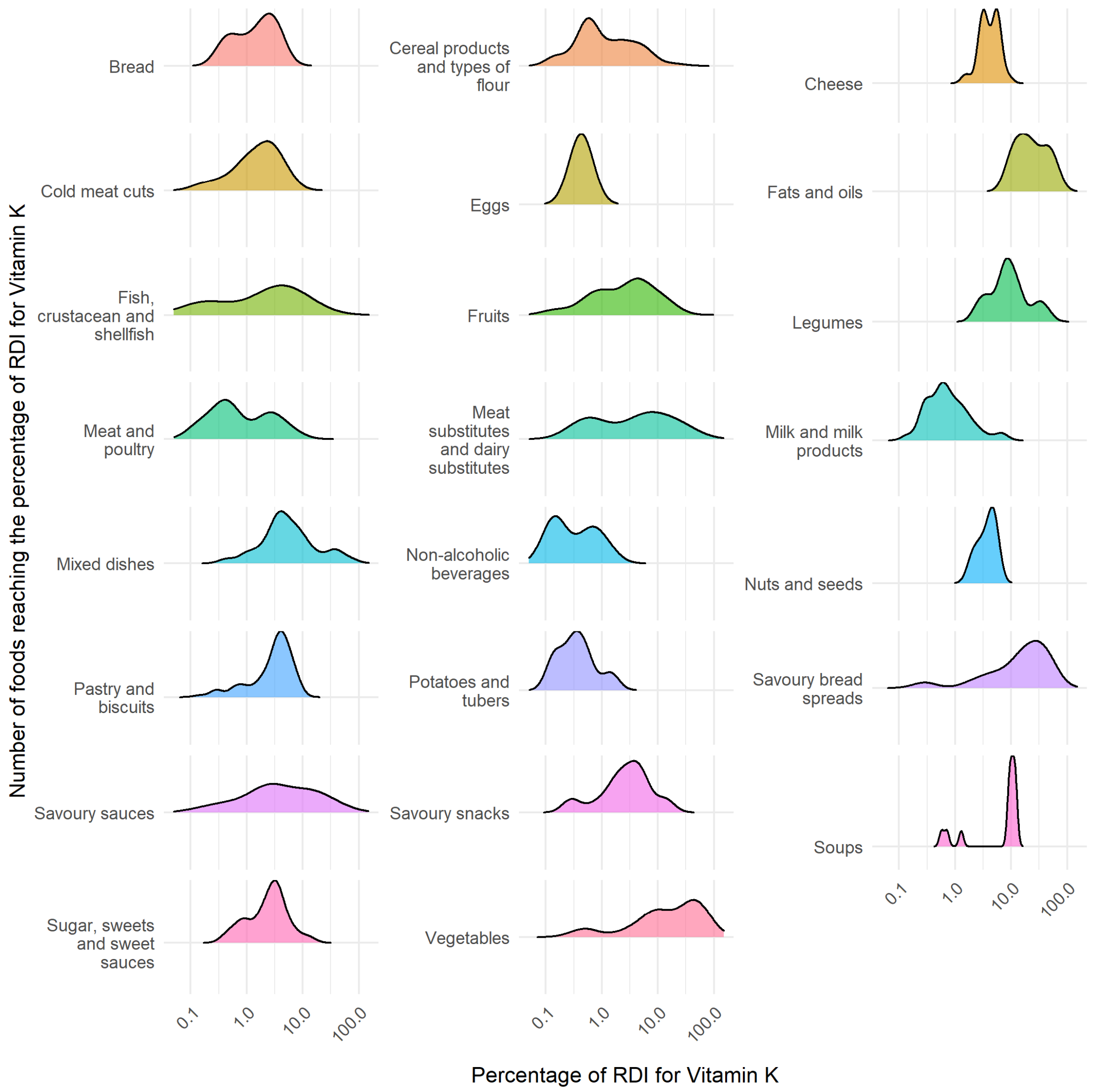

Bread
Cereal products and types of flour
Cheese
Cold meat cuts
Eggs
Fats and oils
Fish, crustacean and shellfish
Fruits
Legumes
Meat and poultry
Meat substitutes and dairy substitutes
Milk and milk products
Mixed dishes
Non-alcoholic beverages
Nuts and seeds
Pastry and biscuits
Potatoes and tubers
Savoury bread spreads
Savoury sauces
Savoury snacks
Soups
Sugar, sweets and sweet sauces
Vegetables
0.1
1.0
10.0
100.0
Number of foods reaching the percentage of RDI for Vitamin K
Percentage of RDI for Vitamin K

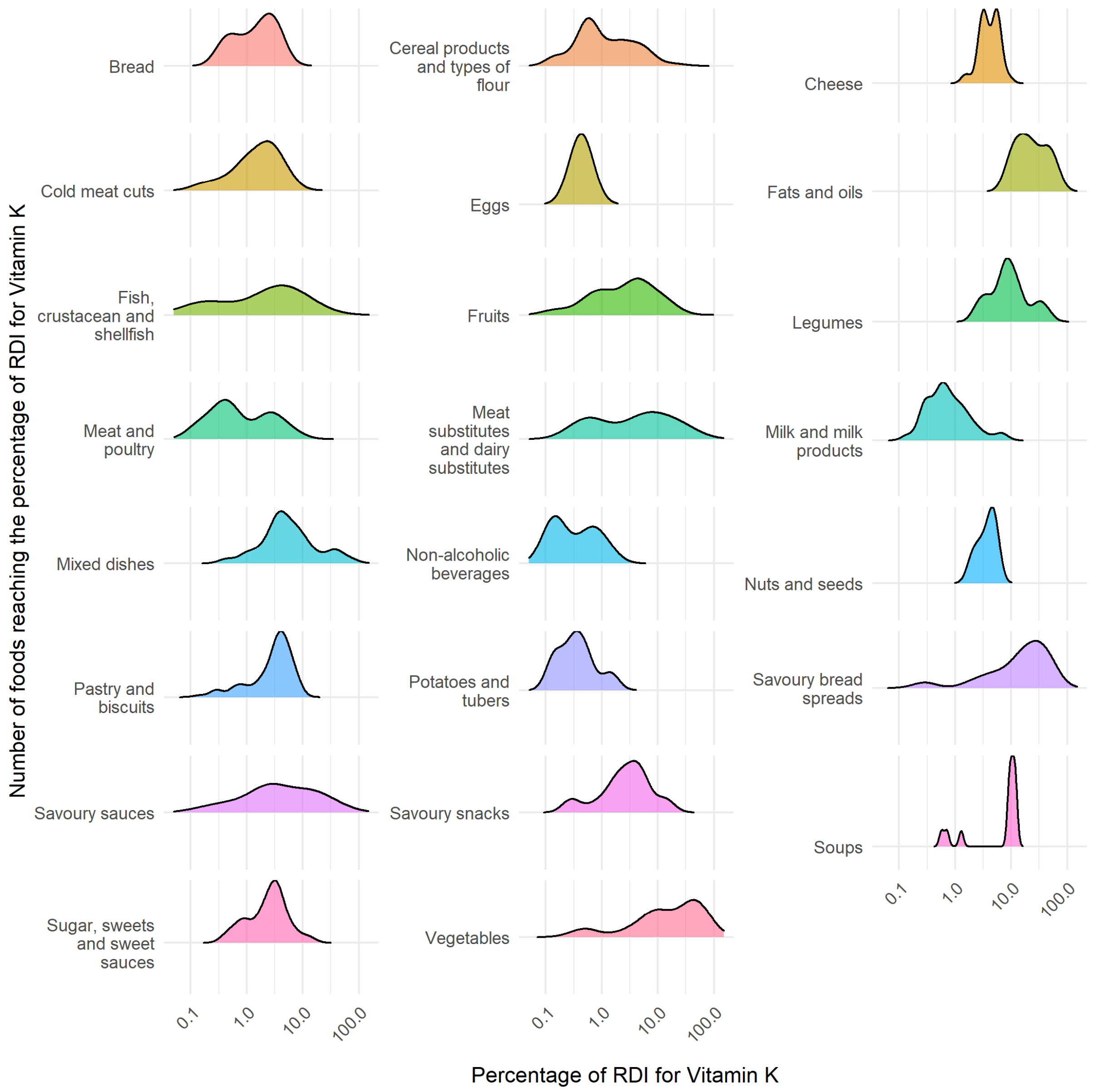

Figure S16. Foods contributing to Reference Daily Intake (RDI) of Vitamin K per 100 gram for each food group; coloured area indicates the amount of foods providing the subsequent percentage of RDI.

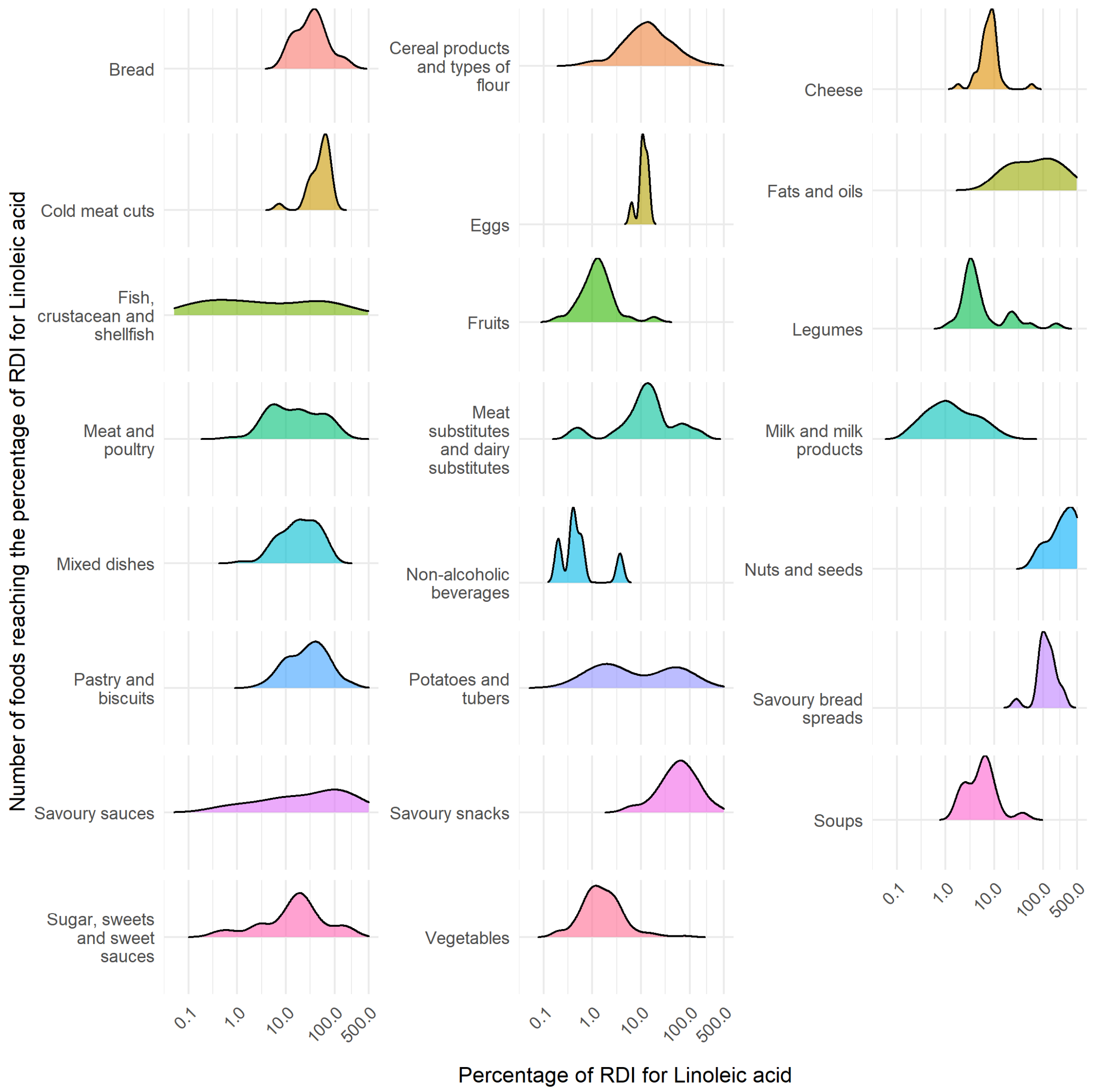

Figure S17. Foods contributing to Reference Daily Intake (RDI) of Linoleic acid per 100 gram for each food group; coloured area indicates the amount of foods providing the subsequent percentage of RDI.

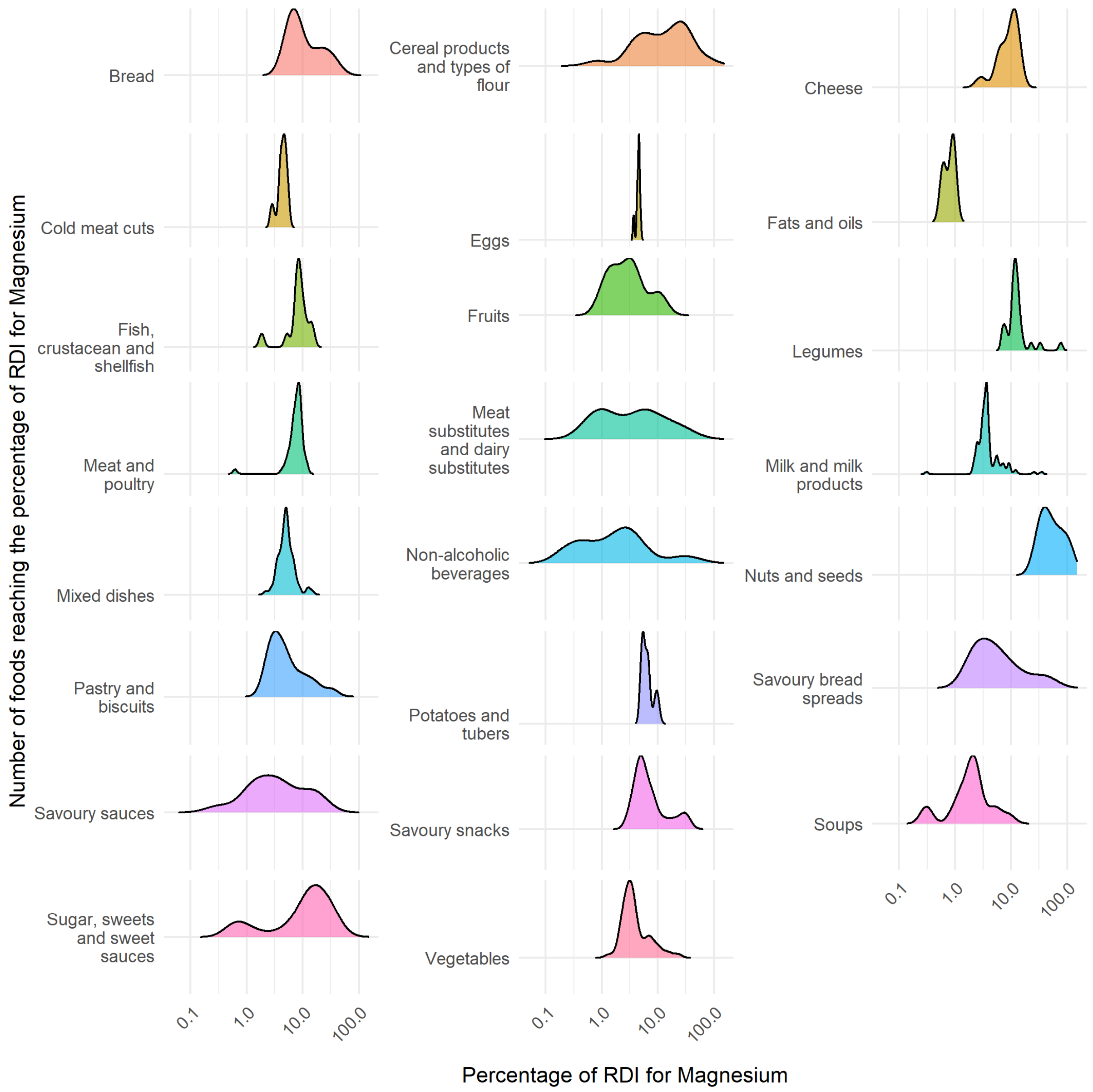

Figure S18. Foods contributing to Reference Daily Intake (RDI) of Magnesium per 100 gram for each food group; coloured area indicates the amount of foods providing the subsequent percentage of RDI.

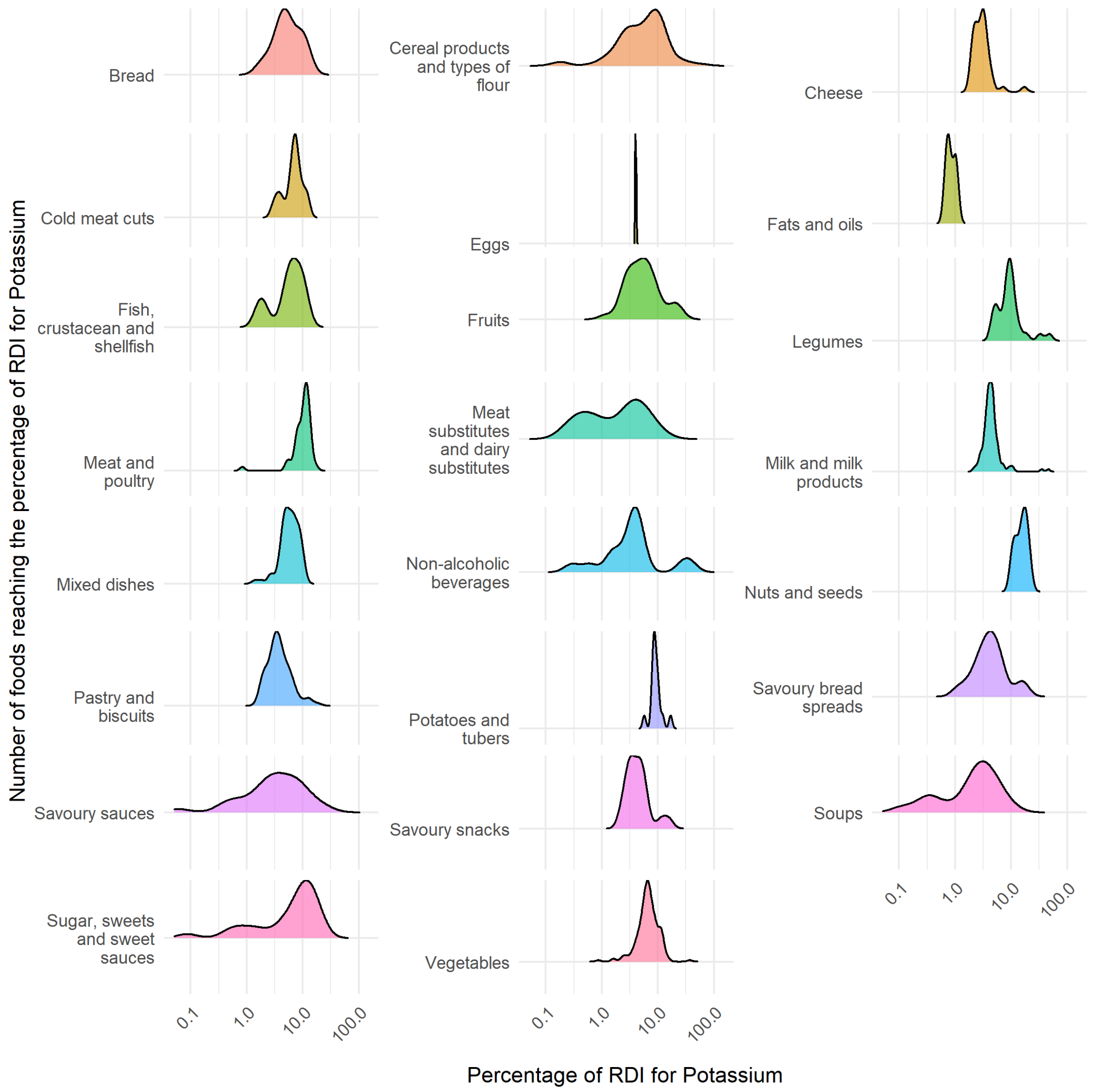

Figure S19. Foods contributing to Reference Daily Intake (RDI) of Potassium per 100 gram for each food group; coloured area indicates the amount of foods providing the subsequent percentage of RDI.

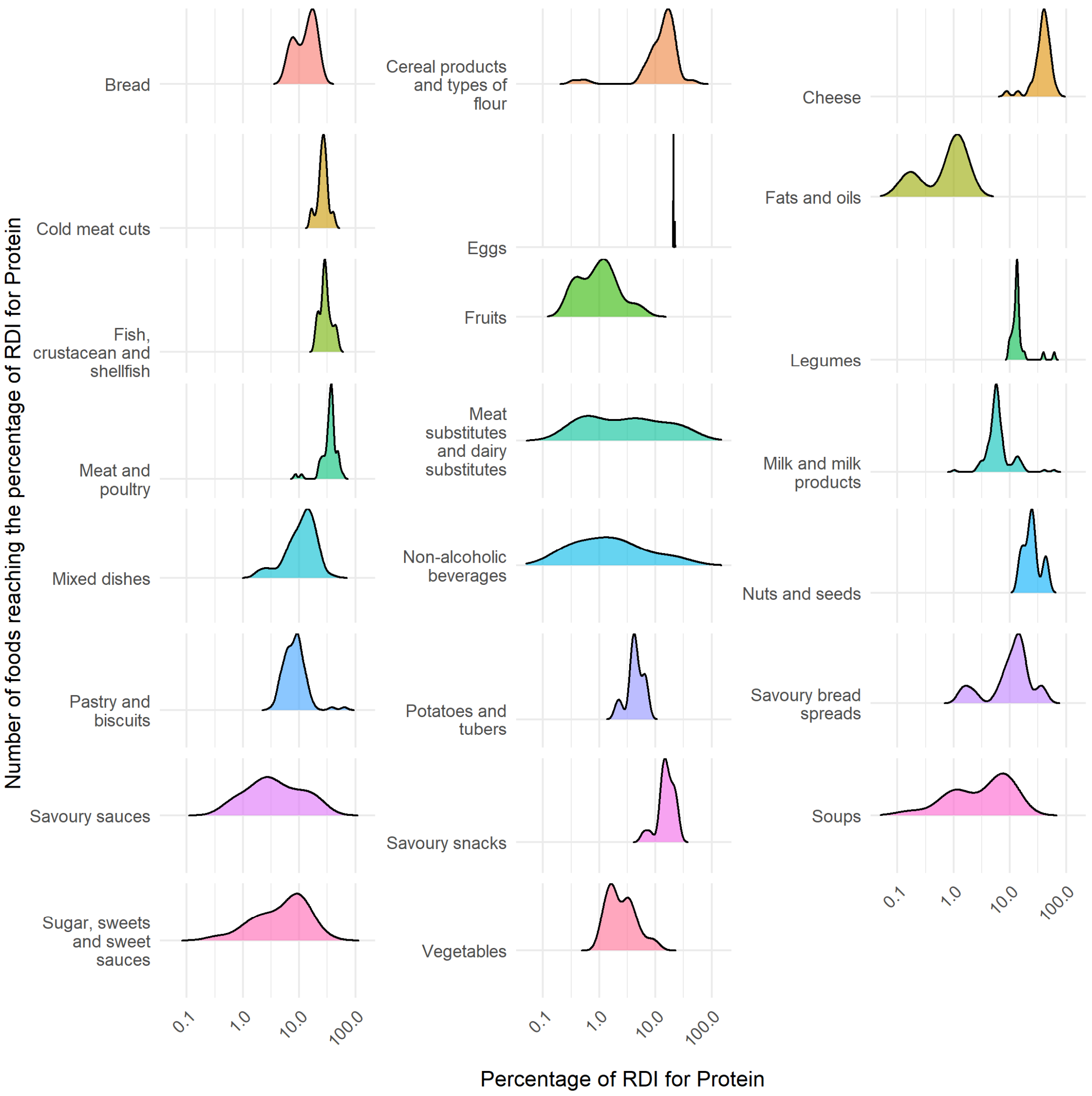

Figure S20. Foods contributing to Reference Daily Intake (RDI) of Protein per 100 gram for each food group; coloured area indicates the amount of foods providing the subsequent percentage of RDI.

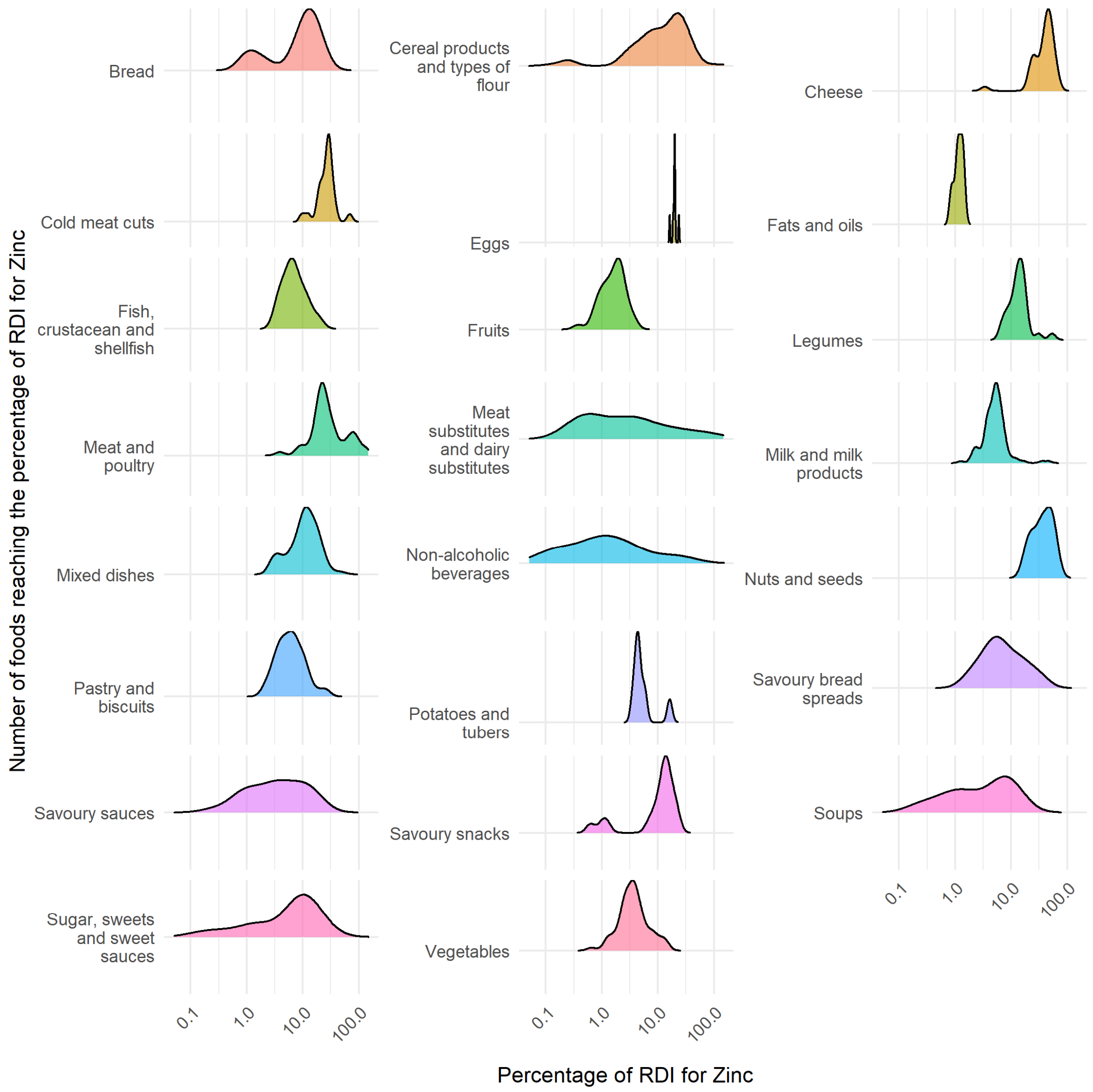

Figure S21. Foods contributing to Reference Daily Intake (RDI) of Zinc per 100 gram for each food group; coloured area indicates the amount of foods providing the subsequent percentage of RDI.

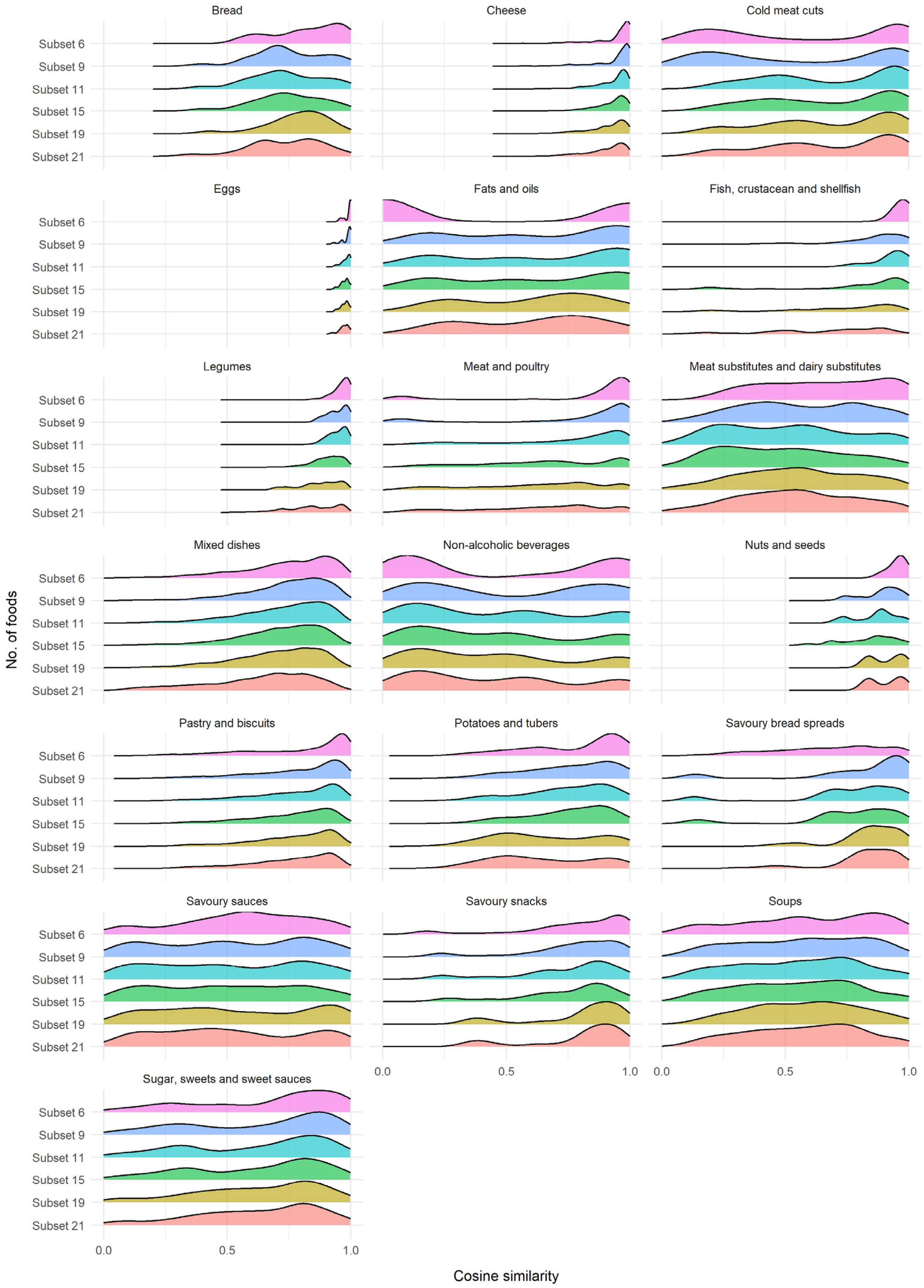

Figure S22. Cosine similarity distribution per food group for each of the six nutrient subsets for intra food category comparisons; coloured area in each graph represents the number of food combinations. Food groups already presented in the manuscript Figure 5 were omitted from this figure.

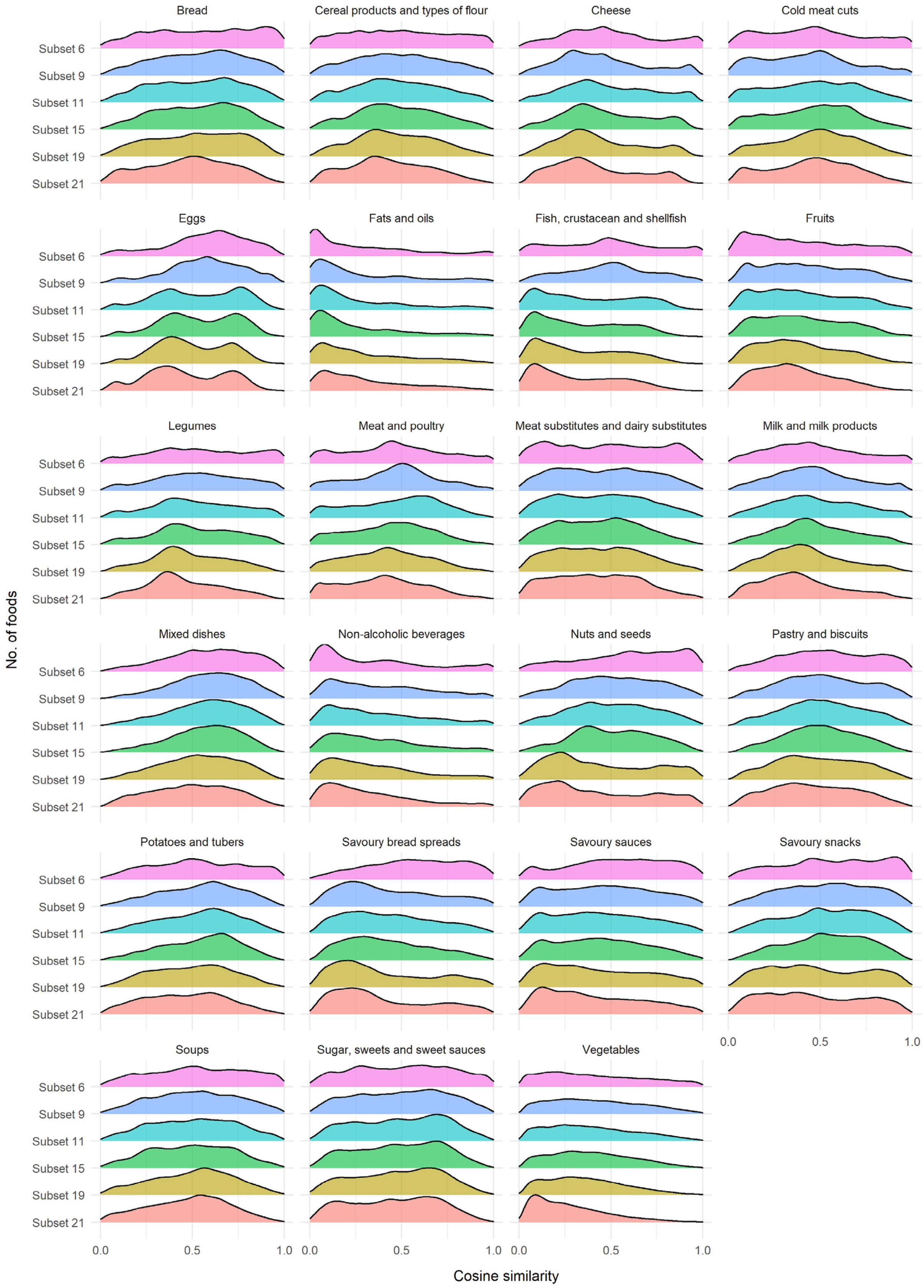

Figure S23. Cosine similarity distribution per food group for each of the six nutrient subsets for inter food category comparisons; coloured area in each graph represents the number of food combinations.

Table S3. Nutrient content per 100 grams for food items included in Figure 6, sourced from the Dutch nutrient content database (NEVO)[1]

Nutrient [unit]	Milk semi-skimmed	Peanut butter	Brown beans	Fish fingers	Soya drink	Salami sausage	Chicken fillet	Mozzarella cheese	Chicken egg	Almonds (unsalted)
Protein [g]	3.4	22.5	6.5	13	3.4	17.8	23.3	18.7	12.3	25.4
Fiber [g]	0	8	7.9	0.8	0.6	0.7	0	0	0	10.2
Potassium [mg]	160	575	285	179	150	249	351	145	145	668
Calcium [mg]	123	45	50	26	17	29	6	160	57	248
Magnesium [mg]	12	145	34	29	18	16	30	10	14	232
Iron [mg]	0	1.8	1.6	0.9	0.5	1.5	0.6	0.4	2.3	3
Zinc [mg]	0.41	2.6	0.54	0.62	0.35	2.34	0.65	2.6	1.56	2.85
Iodine [µg]	14.9	0	0.6	110	1	2.5	0	18	31.3	5.7
Vitamin A [µg]	16	0	0	8	0	18	30	283	194	0
Vitamin D [µg]	0	0	0	2.7	0	0.9	0.3	0.2	1.1	0
Vitamin E [mg]	0	12.4	0.1	2.2	0.4	0.5	0.5	0.4	3.1	25.8
Thiamin (B1) [mg]	0.04	0.13	0.06	0.13	0.02	0.27	0.08	0.03	0.07	0.18
Riboflavin (B2) [mg]	0.18	0.1	0.04	0.08	0.02	0.12	0.03	0.27	0.38	0.91
Vitamin B6 [mg]	0.033	0.02	0.022	0.14	0.052	0.165	0.666	0.1	0.089	0.11
Vitamin B12 [µg]	0.45	0	0	1	0	0.92	0.2	1.7	1.49	0
Niacin (B3) [mg]	0.1	6.2	0.4	3.9	0.2	5.5	11.4	0.4	0	3.4
Folate (B9) [µg]	6.5	81	18.2	15	13.9	2.5	8	20	81.5	93.1
Vitamin C [mg]	1	0	0	0	0	9	0	0	0	0
Vitamin K1 [µg]	0.3	16	6	2.3	4.8	2.3	0	1	0.3	0
Linoleic acid (LA) [g]	0.02	12.8	0.15	1.99	0.9	3.57	0.16	0.26	0.52	13.79
α-linolenic acid (ALA) [g]	0.01	0.73	0.21	0.15	0.12	0.25	0.01	0.09	0.01	0

[1] RIVM NEVO-Online Versie 2025/9.0 2025. https://nevo-online.rivm.nl/

Table S4. The highest 50 cosine similarity values for combinations of foods falling into different food groups.

Food A	Food A Group	Food B	Food B Group	Cosine Similarity
Salad dressing vinaigrette	Savoury sauces	Oil vegetable av	Fats and oils	1.000
Milk semi-skimmed	Milk and milk products	Sauce w semi-skimmed milk and corn starch	Savoury sauces	1.000
Kefir	Milk and milk products	Sauce w semi-skimmed milk and corn starch	Savoury sauces	1.000
Salad cucumber	Savoury bread spreads	Fritter apple	Pastry and biscuits	0.999
Apple turnover w puff pastry wo butter	Pastry and biscuits	Puff pastry av baked	Cereal products and types of flour	0.999
Salad cucumber	Savoury bread spreads	Fritter banana	Pastry and biscuits	0.999
Sauce based on vegetable stock/milk prepared wo fat	Savoury sauces	Blancmange home-made w semi-sk milk	Milk and milk products	0.999
Salad dressing honey/mustard	Savoury sauces	Oil vegetable av	Fats and oils	0.998
Kale curly w mashed potato wo meat prepared	Mixed dishes	Onion Welsh raw	Vegetables	0.998
Kale curly w mashed potato wo meat prepared	Mixed dishes	Onion Welsh boiled	Vegetables	0.998
Topping dessert w sugar whipped KlopKlop	Milk and milk products	Sauce w semi-skimmed milk and corn starch	Savoury sauces	0.998
Kale curly boiled	Vegetables	Kale curly w mashed potato wo meat prepared	Mixed dishes	0.998
Kale curly glass	Vegetables	Kale curly w mashed potato wo meat prepared	Mixed dishes	0.998
Gherkins sweet pickled	Vegetables	Kale curly w mashed potato wo meat prepared	Mixed dishes	0.998
Peanut butter	Savoury bread spreads	Peanut sauce homemade w water w fat	Savoury sauces	0.998
Cucumber sliced pickled glass	Vegetables	Kale curly w mashed potato wo meat prepared	Mixed dishes	0.998

Sauce w semi-skimmed milk and corn starch	Savoury sauces	Blancmange home-made w semi-sk milk	Milk and milk products	0.998
Milk semi-skimmed	Milk and milk products	Sauce based on vegetable stock/milk prepared wo fat	Savoury sauces	0.998
Kefir	Milk and milk products	Sauce based on vegetable stock/milk prepared wo fat	Savoury sauces	0.998
Endive raw	Vegetables	Kale curly w mashed potato wo meat prepared	Mixed dishes	0.998
Butter cream	Fats and oils	Sauce butter-	Savoury sauces	0.997
Salad cucumber	Savoury bread spreads	Pesto red	Savoury sauces	0.997
Milk whole	Milk and milk products	Coffee latte macchiato freshly made	Non-alcoholic beverages	0.997
Coffee latte macchiato freshly made	Non-alcoholic beverages	Milk whole lactose free	Milk and milk products	0.997
Topping dessert w sugar whipped KlopKlop	Milk and milk products	Sauce based on vegetable stock/milk prepared wo fat	Savoury sauces	0.997
Butter unsalted	Fats and oils	Sauce butter-	Savoury sauces	0.997
Porridge milk w wheat flour white Lammetjespap	Milk and milk products	Coffee latte macchiato freshly made	Non-alcoholic beverages	0.997
Roti Surinam pancake	Cereal products and types of flour	Deep fried legumes Bara Surinam	Savoury snacks	0.997
Spinach tinned	Vegetables	Kale curly w mashed potato wo meat prepared	Mixed dishes	0.997
Fritter banana	Pastry and biscuits	Pesto red	Savoury sauces	0.997
Butter salted	Fats and oils	Sauce butter-	Savoury sauces	0.997
Spinach frozen boiled	Vegetables	Kale curly w mashed potato wo meat prepared	Mixed dishes	0.996
Kale curly frozen boiled	Vegetables	Kale curly w mashed potato wo meat prepared	Mixed dishes	0.996
Spinach boiled	Vegetables	Kale curly w mashed potato wo meat prepared	Mixed dishes	0.996
Coffee iced	Non-alcoholic beverages	Milk whole lactose free	Milk and milk products	0.996

Milk skimmed	Milk and milk products	Sauce based on vegetable stock/milk prepared wo fat	Savoury sauces	0.996
Pate	Cold meat cuts	Liver pork raw	Meat and poultry	0.996
Spinach creamed frozen boiled	Vegetables	Kale curly w mashed potato wo meat prepared	Mixed dishes	0.996
Milk whole	Milk and milk products	Coffee iced	Non-alcoholic beverages	0.996
Endive frozen unprepared	Vegetables	Kale curly w mashed potato wo meat prepared	Mixed dishes	0.996
Roti Surinam pancake	Cereal products and types of flour	Croutons	Bread	0.996
Endive boiled	Vegetables	Kale curly w mashed potato wo meat prepared	Mixed dishes	0.996
Cress garden raw	Vegetables	Kale curly w mashed potato wo meat prepared	Mixed dishes	0.996
Salad cucumber	Savoury bread spreads	Tapenade olive	Savoury sauces	0.996
Porridge milk w wheat flour white Lammetjespap	Milk and milk products	Coffee iced	Non-alcoholic beverages	0.996
Spinach raw	Vegetables	Kale curly w mashed potato wo meat prepared	Mixed dishes	0.996
Lettuce butterhead raw	Vegetables	Kale curly w mashed potato wo meat prepared	Mixed dishes	0.996
Plant-based alternative to cream based on soya	Meat substitutes and dairy substitutes	Salad dressing honey/mustard	Savoury sauces	0.996
Peanut butter w peanut pieces	Savoury bread spreads	Peanut sauce homemade w water w fat	Savoury sauces	0.996
Tapenade olive	Savoury sauces	Fritter apple	Pastry and biscuits	0.996

Table S5. The lowest 50 cosine similarity values for combinations of foods falling into the same food group.

Food A	Food B	Food Group	Cosine Similarity
Spread duo wo chocolate	Syrup apple fortified w iron	Sugar, sweets and sweet sauces	0.008
Water >100 mg calcium p litre	Fruit juice drink ACE	Non-alcoholic beverages	0.010
Syrup apple rinse	Spread duo wo chocolate	Sugar, sweets and sweet sauces	0.011
Spread duo wo chocolate	Icing (sugar)	Sugar, sweets and sweet sauces	0.012
Mushroom boiled	Kale curly glass	Vegetables	0.012
Kale curly boiled	Mushroom boiled	Vegetables	0.013
Water 50-100 mg calcium p litre	Fruit juice drink ACE	Non-alcoholic beverages	0.013
Mushroom boiled	Kale curly frozen boiled	Vegetables	0.014
Water av	Fruit juice drink ACE	Non-alcoholic beverages	0.014
Water 0-50 mg calcium p litre	Fruit juice drink ACE	Non-alcoholic beverages	0.015
Salad dressing vinaigrette	Salsa tomato dip	Savoury sauces	0.015
Kale curly glass	Garlic cooked wo fat	Vegetables	0.017
Mushroom boiled	Endive frozen unprepared	Vegetables	0.017
Kale curly boiled	Garlic cooked wo fat	Vegetables	0.018
Kale curly frozen boiled	Garlic cooked wo fat	Vegetables	0.018
Carrot tinned	Peppers pickled canned/glass	Vegetables	0.019
Mushroom tinned	Kale curly frozen boiled	Vegetables	0.019
Carrot winter boiled	Peppers pickled canned/glass	Vegetables	0.020
Mushroom tinned	Kale curly glass	Vegetables	0.021
Spinach frozen boiled	Garlic cooked wo fat	Vegetables	0.021
Kale curly boiled	Mushroom tinned	Vegetables	0.021
Fruit drink conc diluted 1 to 7	Fruit juice drink ACE	Non-alcoholic beverages	0.023
Mushroom tinned	Endive frozen unprepared	Vegetables	0.023
Mushroom boiled	Spinach tinned	Vegetables	0.023
Mushroom boiled	Carrot tinned	Vegetables	0.024
Endive boiled	Mushroom boiled	Vegetables	0.024
Endive frozen unprepared	Garlic cooked wo fat	Vegetables	0.024
Mushroom boiled	Spinach frozen boiled	Vegetables	0.025
Sauce based on vegetable stock/milk prepared wo fat	Salad dressing vinaigrette	Savoury sauces	0.026

Sauce w semi-skimmed milk and corn starch	Salad dressing vinaigrette	Savoury sauces	0.026
Spinach tinned	Garlic cooked wo fat	Vegetables	0.027
Mushroom boiled	Carrot winter boiled	Vegetables	0.027
Carrot tinned	Garlic cooked wo fat	Vegetables	0.027
Mushroom raw	Carrot tinned	Vegetables	0.027
Endive boiled	Garlic cooked wo fat	Vegetables	0.028
Spinach boiled	Garlic cooked wo fat	Vegetables	0.029
Spread duo wo chocolate	Syrup apple-pear	Sugar, sweets and sweet sauces	0.029
Carrot winter raw	Peppers pickled canned/glass	Vegetables	0.029
Carrot tinned	Garlic raw	Vegetables	0.029
Spinach creamed frozen boiled	Garlic cooked wo fat	Vegetables	0.029
Carrot tinned	Sweet pepper yellow boiled	Vegetables	0.030
Mushroom raw	Kale curly frozen boiled	Vegetables	0.030
Endive boiled	Mushroom tinned	Vegetables	0.030
Kale curly raw	Garlic cooked wo fat	Vegetables	0.031
Mushroom tinned	Carrot tinned	Vegetables	0.031
Carrot boiled av	Peppers pickled canned/glass	Vegetables	0.031
Carrot tinned	Sweet pepper yellow raw	Vegetables	0.031
Turnip tops boiled	Garlic cooked wo fat	Vegetables	0.032
Mushroom boiled	Cress garden raw	Vegetables	0.032
Plant-based alternative to cream based on soya	Drink coconut w sugar fortified w calcium and vitamins	Meat substitutes and dairy substitutes	0.032
